# Retrospective harm benefit analysis of pre-clinical animal research for six treatment interventions

**DOI:** 10.1371/journal.pone.0193758

**Published:** 2018-03-28

**Authors:** Pandora Pound, Christine J. Nicol

**Affiliations:** 1 Population Health Sciences, University of Bristol, Canynge Hall, Bristol, United Kingdom; 2 School of Veterinary Science, University of Bristol, Langford House, Langford, United Kingdom; Universidade do Porto Instituto de Biologia Molecular e Celular, PORTUGAL

## Abstract

**Background:**

The harm benefit analysis (HBA) is the cornerstone of animal research regulation and is considered to be a key ethical safeguard for animals. The HBA involves weighing the anticipated benefits of animal research against its predicted harms to animals but there are doubts about how objective and accountable this process is.

**Objectives:**

i. To explore the harms to animals involved in pre-clinical animal studies and to assess these against the benefits for humans accruing from these studies; ii. To test the feasibility of conducting this type of retrospective HBA.

**Methods:**

Data on harms were systematically extracted from a sample of pre-clinical animal studies whose clinical relevance had already been investigated by comparing systematic reviews of the animal studies with systematic reviews of human studies for the same interventions (antifibrinolytics for haemorrhage, bisphosphonates for osteoporosis, corticosteroids for brain injury, Tirilazad for stroke, antenatal corticosteroids for neonatal respiratory distress and thrombolytics for stroke). Clinical relevance was also explored in terms of current clinical practice. Harms were categorised for severity using an expert panel. The quality of the research and its impact were considered. Bateson’s Cube was used to conduct the HBA.

**Results:**

The most common assessment of animal harms by the expert panel was ‘severe’. Reported use of analgesia was rare and some animals (including most neonates) endured significant procedures with no, or only light, anaesthesia reported. Some animals suffered iatrogenic harms. Many were kept alive for long periods post-experimentally but only 1% of studies reported post-operative care. A third of studies reported that some animals died prior to endpoints. All the studies were of poor quality. Having weighed the actual harms to animals against the actual clinical benefits accruing from these studies, and taking into account the quality of the research and its impact, less than 7% of the studies were permissible according to Bateson’s Cube: only the moderate bisphosphonate studies appeared to minimise harms to animals whilst being associated with benefit for humans.

**Conclusions:**

This is the first time the accountability of the HBA has been systematically explored across a range of pre-clinical animal studies. The regulatory systems in place when these studies were conducted failed to safeguard animals from severe suffering or to ensure that only beneficial, scientifically rigorous research was conducted. Our findings indicate a pressing need to: i. review regulations, particularly those that permit animals to suffer severe harms; ii. reform the processes of prospectively assessing pre-clinical animal studies to make them fit for purpose; and iii. systematically evaluate the benefits of pre-clinical animal research to permit a more realistic assessment of its likely future benefits.

## Introduction

Many countries require research projects using animals to be independently evaluated by a competent authority as part of the approval or licensing process. This prospective evaluation often involves weighing the anticipated benefits of the research against its predicted harms to animals. In the European Union (EU) a harm benefit analysis (HBA) is conducted to assess ‘whether the harm to the animals in terms of suffering, pain and distress is justified by the expected outcome’ and whether the research ‘may ultimately benefit human beings, animals or the environment.’[1] (Article 38) The HBA has been a legal requirement in the UK since the Animals (Scientific Procedures) Act 1986. Since then several other countries including Norway, Brazil, Tanzania and Australia have adopted similar provisions.[2] Scientists using animals for research in European Union (EU) member states have been required to conduct a HBA since 2013.[1] Whilst the United States Department of Agriculture Animal Welfare Act does not require a HBA to be performed, the US Institutional Animal Care and use Committee is obliged to weigh the objectives of each study against its potential harms to animals.[2] Elsewhere there appears to be no formal requirement for a HBA, with animal research being conducted according to guiding principles (e.g. Japan), local laws, or subject to approval by ethics committees (e.g. Canada).

There are growing doubts about whether the HBA process is sufficiently consistent and objective, with increasing calls for it to become more transparent, systematic and accountable.[2–11] The UK government’s Animals in Science Committee (ASC), has recently recommended that methods for prospectively assessing harms and benefits should be continually improved and updated and that societal concerns about animal research should be explored and addressed.[12] Public support for animal research is conditional upon the minimisation of harms to animals and upon benefits to humans and other animals;[11] however in the UK at least, only 41% of the public trusts scientists not to cause unnecessary suffering to animals.[13] At present public scrutiny of the HBA process is not possible because although non-technical project summaries of approved license applications are publicly available in the UK[14] and other EU countries, they do not include the severity category of the research and cannot be linked to publications reporting the outcome of that research (due to anonymity). Directive 2010/63/EU (Article 39: 2) requires researchers using non-human primates and /or severe procedures to conduct retrospective assessments of their individual projects[15] but it is unclear whether these will be made publicly available.

Accountability however, can be explored by investigating the outcomes of earlier decisions to approve animal studies, i.e. by finding out what benefits have actually accrued from animal studies that have already been conducted and what these studies actually involved in terms of harms to animals. This type of retrospective HBA might also improve the process of conducting *prospective* HBAs, by suggesting criteria for assessing benefits or by providing a more realistic view of the likely benefit of animal studies based on past experience. Whilst potential benefits might include increased knowledge or safety, our interest here is in the clinical benefit of animal research for humans. However there are challenges involved in this type of retrospective HBA, particularly with regard to determining clinical benefit. The ideal is to use systematic review data (rather than data from single studies) but while systematic reviews of pre-clinical animal studies investigate treatment effects in animals (and scientific rigour) they do not tend to consider relevance for humans. Thus the immediate obstacle to conducting a retrospective HBA of pre-clinical animal research is the lack of systematic data available on clinical benefit.

There have been various attempts to evaluate the clinical benefits of animal research for humans, including consulting physicians for their views[16], historical investigation of drug developments,[17, 18] citation analysis to track the flow of knowledge from the laboratory to the clinic,[19] tracking studies that clearly indicate future clinical application,[20] assessing research ‘payback’[21] and comparing findings from systematic reviews of animal studies with systematic reviews of humans studies for the same interventions. [22] After considering the available options we decided that the latter study, published in 2007 by Perel et al,[22] provided the most suitable data for a retrospective HBA, not only because the study was rigorous and considered a range of treatment interventions but also because the studies reviewed had been conducted sufficiently long ago for their clinical benefits to be assessed.

Our aims were to reanalyse Perel et al’s data to i. explore the actual harms to animals involved in the studies and to assess these against the actual benefits for humans accruing from these studies; and ii. test the feasibility of the retrospective HBA method. To our knowledge this is the first time this type of systematic retrospective HBA has been attempted.

## Methods

### Perel et al’s sample

Perel et al[22] identified 6 interventions for which there was unambiguous systematic review evidence of a treatment effect for humans: corticosteroids for brain injury, antenatal corticosteroids for neonatal respiratory distress, bisphosphonates for osteoporosis, antifibrinolytics for haemorrhage, thrombolytics for stroke and Tirilazad for stroke. Having identified these interventions, they searched for all published and unpublished controlled animal studies for the same 6 interventions, with no restriction by date of publication. To be eligible for inclusion the studies had to report outcomes corresponding to those for which a treatment effect (either positive or negative) had been shown in clinical trials. The authors identified and systematically reviewed 228 animal studies relating to the 6 interventions. They assessed the methodological quality of the animal studies based on measures taken to prevent bias (allocation concealment, blinded assessment of outcome and random allocation to groups) as ‘poor’ for studies in all 6 interventions. Comparing the results from the systematic reviews of animal studies with the systematic reviews of clinical studies they found that two interventions (bisphosphonates, thrombolytics) were concordant, i.e. the findings from the animal studies agreed with the findings from the human studies, one intervention was partially concordant (antenatal corticosteroids) and three were discordant (corticosteroids, Tirilazad and antifibrinolytics). Concordance between animal and human studies suggests that the animal studies represent or model the human condition adequately. Thus concordance provides an indication of clinical relevance, although it does not necessarily imply that the animal studies led directly to human benefit. A limitation is that Perel et al do not state how they selected the 6 interventions from among other potentially relevant interventions, but as our purpose is to test the feasibility of conducting a retrospective HBA (not to obtain a random sample of animal research), their sample is appropriate, particularly since it contains a range of interventions and spread in terms of concordance/ discordance. We searched for the 228 animal studies and noted their citation scores at the time of retrieval (May—June 2015).

### Data extraction on harms

Perel et al did not document animal harms, nor welfare and reported animal numbers only where methodologically relevant. We systematically extracted data on harms, welfare and animal numbers from each of the studies, with a second reviewer conducting independent data extraction on a random sample of 20% of papers (n = 42) to check the accuracy and consistency of the process.

We extracted data on the procedures animals underwent, including use of anaesthesia, paralytic agents or painkillers, post-operative care, how and when animals were killed, any unexpected deaths or events and the species and number of animals used. Every effort was made to correctly document animal numbers (by carefully scrutinising the text and tables) but due to poor reporting estimates occasionally had to be made using all available information. We have indicated where this is the case.

We extracted welfare information where this was available, including any mention of diet, water and housing (i.e. individual / group housing, paddocks, metabolic cages, temperature, lighting). Very rarely information was found on animal stress, purchase, quarantine, transport, handling, breeding, mating and monitoring; this was also extracted. We noted whether studies reported that they had ethical approval or had followed guidelines.

### Expert panel to categorise the severity of harms

A panel of experts from the School of Veterinary Sciences (University of Bristol) was convened to categorise the severity of harms. The panel consisted of 2 professors, 3 senior research fellows and 1 senior lecturer in animal welfare. Five of the panel members have PhDs in animal welfare science, 3 are veterinary surgeons (2 holding the RCVS Diploma in Animal Welfare Science, Ethics and Law) and all are actively engaged in animal welfare research (including pain perception in rats and sheep, assessment of central pain processing in dogs, development of automated tests of laboratory animal welfare, and humane slaughter of farm, laboratory and wild animals).

The EU’s severity classification (Annex VIII) was used since it is employed by the EU regulatory bodies when performing HBAs[1] and because it is similar to the American system; both classify pain, suffering and distress into categories of mild, moderate or severe.[23] The first author ran the scoring workshop and the second author observed. Rather than asking the panel to assess all the studies, members were asked to assess typical procedures for each of the 6 interventions (corticosteroids, thrombolytics, etc.), including a range of actual endpoints. The antifibrinolytic studies were too varied to summarise as they employed markedly different methods of inducing bleeding and aimed to treat a range of different conditions (e.g. haemorrhage, haematuria, gastric haemorrhage, microarterial trauma) so all studies were presented in this case. (The other studies used a more homogenous range of methods and models to treat single conditions.) Panel members were asked to independently categorise the overall impact of the procedures employed for each of the 6 interventions as mild, moderate, or severe. Non-recovery procedures were excluded where this was clear (Box 1).

**Box 1. Severity categorised according to directive 2010/63/EU[1]**

**Mild**

Procedures on animals as a result of which the animals are likely to experience short-term mild pain, suffering or distress, as well as procedures with no significant impairment of the well-being or general condition of the animals.

**Moderate**

Procedures on animals as a result of which the animals are likely to experience short-term moderate pain, suffering or distress, or long-lasting mild pain, suffering or distress, as well as procedures that are likely to cause moderate impairment of the well-being or general condition of the animals.

**Severe**

Procedures on animals as a result of which the animals are likely to experience severe pain, suffering or distress, or long-lasting moderate pain, suffering or distress, as well as procedures that are likely to cause severe impairment of the well-being or general condition of the animals.

**Non-recovery**

Procedures which are performed entirely under general anaesthesia from which the animal shall not recover consciousness.

### Clinical relevance

Benefit was assessed in terms of clinical relevance. Clinical relevance was indicated by: i. concordance / discordance between the findings from systematic reviews of animal and human studies for the same interventions, with concordance suggesting that animal studies model the human condition adequately[22], and ii. current clinical practice relating to the 6 interventions.

### Data analysis

**Fig 1:**
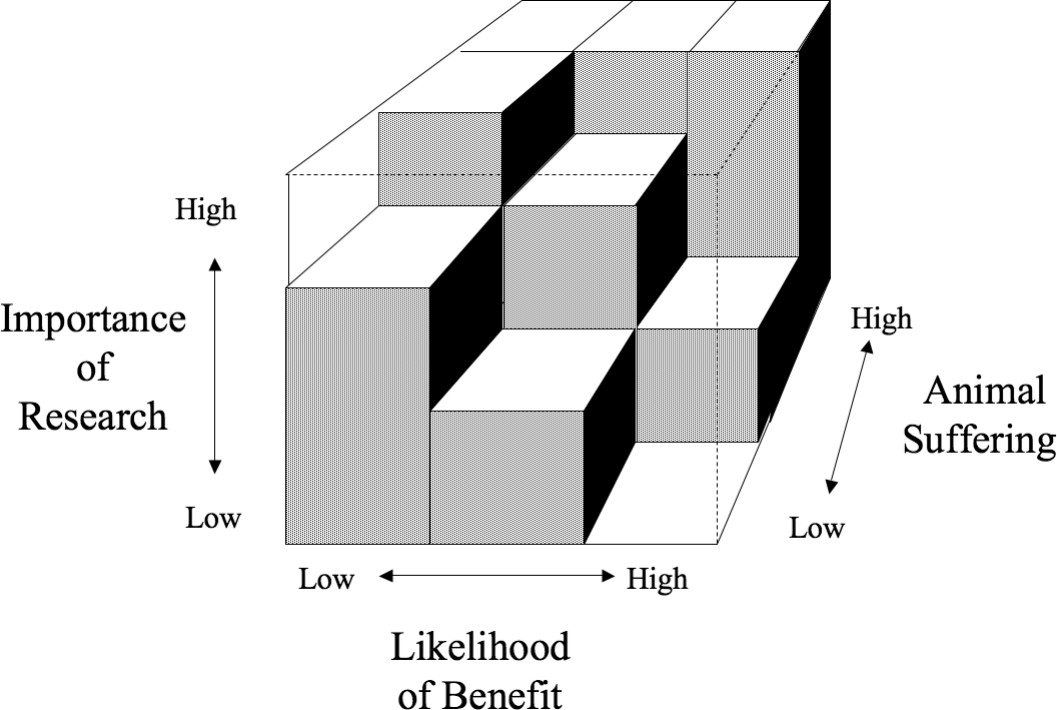
Bateson’s cube.

Extracted textual data were content analysed. Quantitative data were aggregated. The analysis of harms and benefits was guided by Bateson’s Cube[3] (Fig 1). Bateson’s Cube was designed to guide decision-making for authorising individual animal studies but in practice it has proved difficult to use prospectively.[2] We tested its use as a guide to conducting a *retrospective* HBA.

We used the most recent version of Bateson’s Cube which consists of 3 axes assessing animal suffering, the likelihood of benefit and the importance of research. (The axes on the original version of the cube were animal suffering, certainty of medical benefit and quality of research, respectively.) According to Bateson the ‘importance of research’ axis refers to both research quality and conceptual advances unrelated to clinical benefit.[24] For this axis we use Perel et al’s assessments of research quality, but we cannot ascertain whether the studies produced conceptual advances unrelated to clinical benefit. Citation scores, however, are able to indicate the broad (not necessarily clinical) impact of the animal studies and are used as such in this context. Box 2 shows the data used for each of the axes. According to Bateson, if the three assessments fall into the solid part of the cube the project is unacceptable.[3]

**Box 2. Data for the 3 axes of Bateson’s cube**

**Animal suffering axis:** extracted data on harms, expert panel severity classifications

**Likelihood of benefit axis:** concordance between animal and human studies, current clinical practice

**Importance of research axis:** Perel et al’s assessment of research quality, citation scores for the animal studies

Box 3 outlines the terminology used in this paper.

**Box 3. Terminology**

**Interventions:** the 6 different treatments being tested, i.e. antifibrinolytics, thrombolytics etc.

**Studies:** the 228 studies conducted to test the interventions

**Experiments:** one or more experiments may be conducted within individual studies

**Procedures:** the actions performed on animals during experiments, categorised for severity as part of the HBA

**Harms:** the pain, suffering or distress animals may experience during, and/ or as a result of, procedures

**Animal model:** where animals are used to ‘model’, or mimic, human conditions

**Endpoint:** the planned time for completion of the experiment, when animals are killed (NB: these are not predefined humane endpoints)

## Results

### Sample

Two hundred and twelve of the 228 papers reviewed by Perel et al[22] were obtained. Fifteen papers, all on thrombolytic therapy for stroke, could not be traced despite the help of experienced librarians. A further paper was excluded as it was a review. The references and missing papers are listed in S1 File. References to the 228 animal studies.

### Data extraction

Agreement between the 2 independent data extractors was very high for most items (average 93% agreement) except for the item on the number of animals used (79% agreement).

### The studies

The United States was the most common location for the research, followed by Europe and Japan (Table 1). Just over half the studies (52%) were conducted by universities, followed by hospital and university collaborations (17%), pharmaceutical company collaborations (9%), pharmaceutical companies alone (8%) and other institutes and collaborations (13%). They span the years 1967 to 2005, with most studies conducted in the 1990s for all interventions except for the bisphosphonate and thrombolytic studies, of which more were conducted in the 2000s than other decades. The studies involved an estimated 27,149 animals, including rats, mice, guinea pigs, rabbits, pigs, non-human primates (baboons, monkeys, squirrel monkeys), cats, sheep and cows. All studies used animals as models of human conditions, rather than for regulatory purposes.

**Table 1:** Study locations, numbers of animals and publication dates.

	Location	Institution	Publication dates of studies
**Antifibrinolytics** (8 studies)	USA 4; Sweden 2; France 1; Switzerland 1	Universities 4; hospitals 2*; pharmaceutical company 1; hospital/ independent institute collaboration 1 *Total pharmaceutical or pharmaceutical collaboration*: *1 (12*.*5%)*	1967–1997 (no mode, more studies conducted in 1990s than other decades)
**Bisphosphonates** (16 studies)	USA 6; Japan 3; Poland 2; China 2; Italy 2; Brazil 1	Pharmaceutical and hospital / university collaboration 5; pharmaceutical company 4; university 4; hospital 1; hospital/ university collaboration 1; centre for disease control 1 *Total pharmaceutical or pharmaceutical collaboration*: *9 (56%)*	1991–2005 (mode 2001, studies evenly distributed between 1990s and 2000s)
**Corticosteroids** (17 studies)	USA 8; Israel 2; Turkey 2; Sweden 2; Germany 1; Mexico 1; South Korea 1; Taiwan 1	Universities 12; pharmaceutical companies 3; hospital / university collaboration 1; hospital 1 *Total pharmaceutical or pharmaceutical collaboration*: *3 (18%)*	1975–2005 (mode 2005, although more studies conducted in 1990s than other decades)
**Tirilazad** (18 studies)	Germany 7; USA 7; UK 1; Canada 1; Sweden 1; South Korea 1; Japan 1; Switzerland 1; Turkey 1	Universities 14; hospital 1; hospital / university collaboration 2; pharmaceutical / university collaboration 1 *Total pharmaceutical or pharmaceutical collaboration*: *1 (5*.*5%)*	1990–2004 (mode 1994, more studies conducted in 1990s than other decades)
**Antenatal corticosteroids** (56 studies)	USA 47; Australia 7; Japan 3; Canada 2; Sweden 1; Chile 1; Hungary 1; Italy 1; Netherlands 1; Germany 1; Austria 1; Finland 1	Universities 32; Hospitals 4; hospital /university collaboration 17; university / primate centre collaboration 1; pharmaceutical and hospital/ university collaboration 1; veterinary college 1 *Total pharmaceutical or pharmaceutical collaboration*: *1 (2%)*	1971–2004 (mode 1997, more studies conducted in 1990s than other decades)
**Thrombolytics** (97 studies)	USA 63; Japan 18; Germany 14; Canada 4; France 4; Belgium 2; South Korea 1; Turkey 1; Switzerland 1	Universities 45; hospitals 6; hospital / university collaboration 16; pharmaceutical company 9; pharmaceutical and university / hospital collaboration 12; independent institute 7; independent institute and university 1; pharmaceutical and independent institute collaboration 1 *Total pharmaceutical or pharmaceutical collaboration*: *22 (23%)*	1987–2005 (mode 2002, more studies conducted in 2000s than other decades)
**Total** (212 studies)	**USA 135; Europe 53; Japan 25; Canada 7; Australia 7; South Korea 3; China 2; Israel 2; South America 2; Mexico 1; Taiwan 1** Total 238 **	**Universities 111; hospital / university collaboration 37; pharmaceutical and hospital/ university collaboration 19; pharmaceutical 17; hospitals 15; independent institute 7; independent institute and hospital/ university 2; independent institute and pharmaceutical 1; university / primate centre collaboration 1; veterinary college 1; centre for disease control 1** ***Total pharmaceutical/ pharmaceutical collaboration*: *37 (17%)***	**All studies: 1967–2005** **More studies conducted in 1990s than in other decades for all interventions except for the bisphosphonate studies, which were evenly distributed between the 1990s and 2000s, and the thrombolytic studies, of which more were conducted in the 2000s than in other decades**

* Hospitals include medical centres

**Several studies involved international collaborations so country totals are greater than the total number of studies

The animal studies were first published before human studies in the case of antifibrinolytics, bisphosphonates and Tirilazad, at around the same time for antenatal corticosteroids, but *after* human studies in the case of corticosteroids and thrombolytics (Table 2). In the case of bisphosphonates, Tirilazad and thrombolytics, publication of animal studies continued after the systematic reviews of the clinical trials were published.

**Table 2:** Comparison of dates of animal and human studies.

	Publication dates of animal studies	Publication dates of human studies	Date of clinical systematic review/ meta-analysis	Number of animal studies conducted after treatment effect known in humans
**Antifibrinolytics** (8 studies)	1967–1997	1987–1998	1999	None
**Bisphosphonates** (16 studies)	1991–2005	1995–1999	2002	4 studies (1 in 2003; 2 in 2004; 1 in 2005)
**Corticosteroids** (17 studies)	1975–2005	1972–2005	2005	None
**Tirilazad** (18 studies)	1990–2004	1994–1997	2001	3 studies (2 in 2003; 1 in 2004)
**Antenatal corticosteroids** (56 studies)	1971–2004	1972–2002	2006	None
**Thrombolytics** (97 studies)	1987–2005	1981–2002	2004	4 studies (all in 2005)

### Reporting

Reporting of animal harms and welfare was poor (Table 3) and the information reported was basic. Reporting of animal numbers was poor, particularly for studies of antenatal corticosteroids, where foetuses might be studied in ‘batches’, with their tissues and blood ‘pooled’. Thirty two percent of all studies (n = 69) failed to report how animals were killed and 10% (n = 21) did not report when they were killed. Seventy percent (n = 148) failed to report any welfare information and only 3 studies reported post-operative care (one simply mentioned that animals were given post-operative care, one reported use of analgesia and penicillin, one reported that animals were monitored and released to gang cages). Thirteen percent of studies (n = 27) failed to report use of anaesthesia and 97% (n = 206) did not report analgesia use. Half of all studies made no ethical statement. Thirty nine studies (18%) reported additional procedures involving further animals but gave little information on the animal numbers or procedures involved. See S1 Table. Additional procedures involving further animals.

**Table 3:** Reporting (n = studies apart from final column).

	No ethical statement reported	Use of anaesthesia not reported	How animals killed not reported	Time of death (post experiment) not reported	Use of painkillers not reported	No welfare information reported	Total number animals used
**Antifibrinolytics** **(n = 8 studies)**	6 (75%)	0	7 (87%)	3 (37%)	7 (87%)	5 (62%)	668
**Bisphosphonates** **(n = 16)**	8 (50%)	4 (25%)	7 (44%)	0	16 (100%)	2 (12%)	807
**Corticosteroids** **(n = 17)**	13 (76%)	0	5 (29%)	3 (18%)	16 (94%)	9 (53%)	2296**
**Tirilazad** **(n = 18)**	10 (56%)	0	3 (17%)	1 (5%)	16 (89%)	12 (67%)	764***
**Antenatal corticosteroids** **(n = 56)**	38 (68%)	14 (25%) for caesarean section 12 (21%) for neither mother nor foetus	12 (21%) [10 = foetal manner of death unreported, (including 1 maternal), plus 2 = maternal only]	4 (7%) [foetuses] 0 (0%) of 11*	55 (98%) [mothers] 56 (100%) [foetuses]	41(73%) [mothers] 56 (100%) [foetuses]	16,000*** (both mothers and neonates)
**Thrombolytics** **(n = 97)**	30 (31%)	11 (11%)	35 (36%)	10 (10%)	96 (99%)	79 (81%)	6614***
**Total (n = 212)**	**106 (50%)**	**27 (13%)**	**69 (32%)**	**21 (10%)**	**206 (97%)**	**148 (70%)**	27,149***

* Only 11 of the antenatal corticosteroid studies reported that mothers were killed.

** Total number of animals used in corticosteroid studies likely to be an underestimate due to the poor reporting of animals excluded from studies

***Estimated number

### Harms

Much of the data presented below is summarised in Table 3 above and Tables 4, 5 and 6 below. Table 5 summarises the severity assessments. Detailed scoring for each severity assessment is available in S2 Table. Results of expert panel severity classifications.

**Table 4:** Reported methods of killing animals (n = number of studies).

**Antifibrinolytics (n = 8)**	Air emboli: n = 1; not reported: n = 7
**Bisphosphonates (n = 16)**	Exsanguination: n = 4; CO_2_ inhalation: n = 3; cardiac puncture: n = 1; euthanasia agent (‘Tanax’): n = 1; not reported: n = 7
**Corticosteroids (n = 17)**	Decapitation: n = 6; CO_2_ inhalation: n = 1; euthanasia agent (potassium chloride): n = 1; perfusion fixation: n = 1; aorta and pulmonary artery cut: n = 1; rapid freezing (liquid nitrogen): n = 1; left to die: n = 1; not reported: n = 5
**Tirilazad (n = 18)**	Perfusion fixation: n = 8; euthanasia agent (potassium chloride): n = 3; anaesthesia overdose (pentobarbital): n = 1; rapid freezing (liquid nitrogen): n = 1; decapitation: n = 1; exsanguination: n = 1; not reported: n = 3
**Antenatal corticosteroids** **(n = 56)**	***Foetuses*** Tracheal blockage (trachea clamped 15, endotracheal tube plugged / clamped 4): n = 19; anaesthesia overdose (pentobarbital 12; phenobarbital 1): n = 13; left to die: n = 6; euthanasia agent (lidocaine): n = 5; decapitation/severing of cervical cord: n = 5; strangulation: n = 4; suffocation: n = 1. ***Mothers*** *(11/56 studies reported mothers were killed)* Anaesthesia overdose (pentobarbital 4; diethyl ether 1): n = 5; decapitation: n = 2; thoracotomy: n = 1 Not reported (foetal and/or maternal): n = 12
**Thrombolytics (n = 97)**	Perfusion fixation: n = 20; anaesthesia overdose (pentobarbital 17; thiamyl sodium 1; thiopental 2): n = 20; decapitation: n = 8; exsanguination: n = 4; rapid freezing: n = 3; euthanasia agents (‘potassium chloride 3, ‘Terminal’ 1, KAX cocktail 1): n = 5; brains removed: n = 3; C0_2_ inhalation: n = 2; missing: n = 1; not reported: n = 35
Total* (n = 212)	Anaesthesia overdose: n = 39; perfusion fixation: n = 29; decapitation: n = 22; tracheal blockage: n = 19; euthanasia agent: n = 15; exsanguination: n = 9; left to die: n = 7; CO_2_ inhalation: n = 6; rapid freezing: n = 5; miscellaneous (air emboli, aorta and pulmonary artery cut, cardiac puncture, suffocation, thoracotomy): n = 5; strangulation: n = 4; brains removed: n = 3; missing: n = 1; not reported: n = 69

* Studies could use more than one means of death

**Table 5:** Summary results of severity assessment by expert panel.

Animal model	Summary of scorers’ assessments	Final summary
Antifibrinolytics to promote blood clotting and control bleeding	The study of the shortest duration (2-6h) was generally assessed as moderate, with two scoring it as mild. The study lasting 24 hours had a spread of scores from mild, through moderate to severe, two scoring moderate on condition that bleeding was limited and analgesia given. Studies lasting 5–10 days: the first involved cutting the tail under anaesthesia and again produced a spread of scores from mild through moderate to severe. The study that involved rabbits having renal injuries inflicted under anaesthesia with 5–10 day follow up was mostly scored as severe.	Studies scored from mild to severe
Bisphosphonates to slow down /prevent bone loss	Most scorers felt the general model was of moderate severity, but rising to the severe category as the duration of the study increased and if no analgesia was used. One scorer consistently categorised procedures as less severe than other scorers for this study.	Mostly moderate, some severe
Corticosteroids to reduce intracranial pressure after traumatic brain injury	In general this model was scored as severe. In cases where animals were not anaesthetised or only lightly anaesthetised all scorers categorised procedures as severe. Endpoints were from 2h up to 30 days. Most endpoints were categorised as severe except for studies of the shortest duration which two scorers categorised as moderate as long as anaesthesia was used. For studies lasting 2–4 weeks all but one scorer categorised harms as severe.	Mostly severe
Tirilazad to protect brain tissue after stroke	The overall model was categorised as severe. Variations of the model were all categorised as severe, except for one study that reported post-operative analgesia; this was categorised as generally severe still, but potentially as less severe.	Severe
Antenatal corticosteroids to reduce neonatal mortality and morbidity in preterm babies	For the overall model scorers categorised harms to the mother as moderate to severe, depending on use of analgesia/ anaesthesia. For the overall model the scorers categorised harms to the foetus as generally severe, particularly if the foetus survived beyond birth and had no anaesthesia/ analgesia. For the administration of drugs to the mother scorers generally categorised this as moderate to mild. For the administration of drug to neonates after delivery, scorers categorised this as severe, particularly if neonates not anaesthetised. For administration of the drug to the foetus in utero scorers categorised the procedures to the foetus as severe if no anaesthesia was used (otherwise mild), and moderate to severe for the mother. For administration of the drug via the mother this was categorised as moderate to severe for the mother and moderate to severe for the foetus. For neonates having mechanical ventilation after delivery scorers categorised this as severe, or moderate if anaesthesia was used. In terms of endpoints, scorers categorised harms for those killed in utero and at delivery as moderate; as moderate to severe for deaths post-delivery (30 mins to 6 days), with increasing severity scores as time post-delivery increased. Harms to neonates left to die with no endpoint were categorised as severe by 5/6 scorers (animals found dead should automatically score as ‘severe’). Neonates having ventilation were scored as experiencing moderate to severe harms unless they were anaesthetised, with severity scores increasing with the amount of time spent on the ventilator.	Foetuses and neonates: mostly moderate to severe Mothers: mostly moderate to severe. Some maternal procedures scored as mild (e.g. maternal drug administration) but these were within overall models scored as moderate to severe.
Thrombolytics (tissue plasminogen activator, or tPA) to dissolve clot/ improve blood flow after stroke	In general scorers categorised the stroke model as severe. Some commented that if anaesthesia/ analgesia were used and duration of study brief, then studies might score less severely. Scorers categorised studies that induced stroke while animals were awake as severe. Most categorised relatively short term studies (up to 33h) that induced stroke under anaesthesia as severe. Most categorised studies that induced stroke under anaesthesia and then involved repeated MRI scans as severe.	Mostly severe

**Table 6:** Summary of harms.

	Endpoint range	No. studies reporting deaths before endpoint	Noteworthy harms reported	Total no. animals (species)	Average no. animals per study	No. studies reporting welfare information	No. studies reporting ethical statement	Severity classification by expert panel
**Antifibrinolytics** **(8 studies)**	4 hrs– 10 days	0		668 (rats, rabbits, pigs)	83	3 (38%)	2 (25%)	Ranged from mild to severe
**Bisphosphonates** **(16 studies)**	4 weeks– 2 years	1 (6%)		807 (rats, baboons)	50	14 (88%)	8 (50%)	Mostly moderate, some severe
**Corticosteroids** **(17 studies)**	2 hrs– 30 days	9 (53%)	Some animals had no or only light anaesthesia prior to restraint and head injury. Some animals left to die of injuries.	2296 (mice, rats, guinea pigs, cats, monkeys)	135	8 (47%)	4 (24%)	Mostly severe
**Tirilazad** **(18 studies)**	3 hrs– 7 days	3 (17%)	Some iatrogenic deaths. Many animals had repeated daily assessments post-surgery, suggesting cumulative harms	764 (rats, rabbits, cats)	42	6 (33%)	8 (44%)	Severe
**Antenatal corticosteroids** **(56 studies)**	0 hrs– 6 days (neonate)	20 (36%)	Most studies did not report neonatal anaesthesia. Some neonatal upper airway leaks during tracheal tube placement, also pneumothoraces. Some neonates left to die.	16,000, (mothers, neonates) Sheep, rabbits, rats, monkeys, baboons, cows	286	Mothers 15 (27%) Foetuses 0	18 (32%)	Mostly moderate to severe for both mothers and neonates
**Thrombolytics** **(97 studies)**	6 hrs– 2 months	37 (38%)	Some animals had stroke induced while conscious and restrained / paralysed. Many had repeated daily assessments and scans, suggesting cumulative harms.	6614 (rats, rabbits, mice, guinea pigs, squirrel monkeys, baboons)	68	18 (19%)	67 (69%)	Mostly severe

#### Antifibrinolytics for haemorrhage

There were 8 studies of antifibrinolytics (aprotinin, aminocaproic acid and tranexamic acid) using various models of blood loss. Six hundred and sixty eight animals were used (485 rats, 109 rabbits, 74 pigs) with an average 83 animals per study. Anaesthesia was reported in all studies. Four models induced blood loss in rats, rabbits or pigs by cutting the ear or tail, with endpoints ranging from 2 hours to 6 days. Two models induced gastric bleeding in rats, one by pouring hydrochloric acid solution into rats' stomachs and the other by creating mucosal lesions in the stomach. Animals were killed at the end of these experiments or after 6 hours. Two models induced blood loss through surgical injury to the internal organs: the first inflicted renal injuries on rabbits by stabbing, crushing, cutting or punch biopsy, with rabbits followed up for 5–10 days, during which they were housed individually in metabolic cages. The second inflicted liver injuries on pigs by applying a clamp to the lobes of the liver. This study reported the use of restraints and paralytics in addition to painkillers and anaesthesia, with the endpoint either death or four hours. Only one study reported how animals were killed and half (n = 4) reported the experimental endpoint. No deaths before endpoint were reported. It was seldom clear how long anaesthesia was maintained for and consequently it was difficult to determine what animals experienced, or for how long. No unexpected events were reported. Only one study reported using painkillers. Three studies (38%) reported welfare information (access to food and water / metabolic cage use). None gave details of post-operative care. Six (75%) did not make an ethical statement.

These studies were mostly scored as moderate or mild, except for the study that involved renal injuries to rabbits with endpoints of up to 10 days, which was mostly scored as severe. Two studies may have been non-recovery but as this was unclear they were scored, mostly as severe.

#### Bisphosphonates for osteoporosis

There were 16 studies of bisphosphonates (mainly alendronate), involving 807 animals (56 baboons, 751 rats) with an average 50 animals per study. The model consisted of ovariectomy followed by drug treatment (usually by oral gavage). Some animals had additional procedures at regular intervals, such as blood and urine tests, bone mineral density measurements, scans and x-rays. Twelve studies (75%) reported using anaesthesia for ovariectomies and/ or additional procedures. Nine studies (56%) reported how animals were killed. All studies reported endpoints, ranging from 4 weeks to 2 years. One study reported deaths prior to endpoint. No unexpected events were reported. No studies reported using paralytic agents. None reported using painkillers. Fourteen studies (88%) reported welfare information (housing/ metabolic cage use/ temperature and lighting /diet and access to water). None gave details of post-operative care. Half made an ethical statement.

This model was mostly scored as moderate, rising to the severe category as the duration of studies increased.

#### Corticosteroids for brain injury

There were 17 studies of corticosteroids (mainly methylprednisolone, dexamethasone, betamethasone), involving 2296 animals (1163 mice, 863 rats, 210 guinea pigs, 31 monkeys, 29 cats), with an average 135 animals per study. The model involved inflicting brain injury on animals and testing the effect of corticosteroids on recovery. The most common way of inducing brain injury (14 studies) was to use stunners or devices designed to drop weights or protruding rods onto restrained animals’ heads. Some animals had steel caps fitted to their skulls, or holes drilled in their skulls prior to injury. In two studies injury was inflicted during brain surgery and in another monkeys were attached to a sled that crashed at speed. In 3 studies animals were not anaesthetised and in 2 studies only lightly anaesthetised. No studies reported using painkillers; one reported that analgesics ‘appeared unnecessary’. No studies reported using paralytics. Animals were reported to die upon impact in 8 studies (47%), accounting for an estimated 10–18% of animals in these studies. One study reported post-operative wound infections in 2 animals. Animals were observed for varying lengths of time post injury and some were tested for neurological status and grip using a string test. Fourteen studies (82%) reported endpoints, ranging from 2 hours to 30 days. Nine studies (53%) reported deaths prior to endpoint. Twelve studies (71%) reported how animals were killed; in one study animals were left to die of their injuries. Eight studies (47%) gave welfare information (diet/ access to water). One study mentioned post-operative care. Most studies (76%) made no ethical statement.

This model was mostly scored as severe. All scorers categorised procedures as severe where animals had no or only light anaesthesia. Most endpoints were categorised as severe except for studies of the shortest duration which two scorers categorised as moderate as long as anaesthesia was used. For studies lasting 2–4 weeks all but one scorer categorised harms as severe.

#### Tirilazad for stroke

There were 18 studies of Tirilazad, involving an estimated 764 animals (25 cats, 111 rabbits, at least 628 rats), with an average of at least 42 animals per study. The model involved an operation under anaesthesia to occlude the middle cerebral artery (MCA) and subsequent testing of the effect of Tirilazad on recovery. During the operation probes and monitors could be placed on the brain. In some studies hypothermia was also induced. Seven studies (39%) occluded the MCA using monofilament. Animals were usually given Tirilazad intra-peritoneally or intravenously at various time points (up to 24h) post-operatively. Anaesthesia was reported in all 18 studies. Two studies reported using analgesia. One reported using a paralytic agent.

There were 2 broad categories of experiment: i. animals had an operation to occlude the MCA, were given Tirilazad and killed up to 24 hours post-operatively. These experiments tended to involve more surgical procedures; ii. animals had an operation to occlude the MCA, were given Tirilazad and then had daily assessments, with death up to 3 days post-operatively, or at 7 days. Post-operative assessments included MRI scans at 24 hours (under anaesthesia), attempts to arouse animals using tactile and painful stimulation up to 24h post-operatively and daily neurological assessments using methods that included pulling animals’ tails and pushing them to test resistance.

Fifteen studies (83%) reported how animals were killed. Seventeen (94%) reported endpoints, ranging from 4 hours to 7 days. Three (17%) reported deaths before endpoint. In one study involving craniectomy and removal of the eye, 5 animals died up to 2 days post-operatively. Six of the 7 studies that occluded the MCA using a monofilament reported that animals suffered vessel perforation and consequent subarachnoid haemorrhage; 29 animals were ‘excluded’ for this reason. Information relating to welfare was given in 6 studies (33%), mainly relating to pre-operative fasting. One study reported post-operative care, noting that animals were given analgesia and penicillin. Ten studies (56%) made no ethical statement.

All variations of the model were categorised as severe. A variation that reported post-operative analgesia was still categorised as severe, but as potentially ‘less severe’.

#### Antenatal corticosteroids for neonatal respiratory distress

There were 56 studies of antenatal corticosteroids (mainly betamethasone, dexamethasone, hydrocortisone) involving an estimated 16,000 animals (2,665 mothers and 13,335 neonates). Of the mothers 1057 were sheep, 727 rats, 699 rabbits, 117 monkeys, 45 cows and 20 baboons. The average number of animals used per study (both mothers and neonates) was estimated at 286. One or more of the following methods were used to administer antenatal corticosteroids: i. administration to pregnant mothers over several weeks before preterm delivery of their neonates. ii. administration to foetuses in utero via injection, laparotomy, intra-amniotic injection or ultrasound-guided foetal injection. iii. administration to foetuses in utero, where mothers were given a hysterotomy and catheters (including tracheal catheters) were placed in foetuses, passing from the uterus through an incision in the mother to her flanks, with drugs delivered for up to 14 days and tracheal fluids withdrawn from some foetuses. iv. administration to neonates after delivery via injection or an endotracheal tube.

Mothers had caesarean section (CS) for preterm delivery of their neonates, after which their neonates were removed. Thirty five studies (62%) reported maternal anaesthesia for CS delivery. Fourteen studies (25%) did not report anaesthesia for CS delivery and 2 studies (4%) reported that no anaesthesia was used (one used sedation only and another reported stretching animals, covering their eyes and removing stitches from a previous operation to deliver neonates). In 5 studies (9%) the mother was killed prior to CS. In addition to CS, further procedures for mothers could include ultrasound scans, amniocentesis, hysterotomy, foetal injections, laparotomy, blood sampling and administration of antibiotics or progesterone. Maternal anaesthesia was reported for additional procedures in 13 studies but not in 11 studies where similar procedures were conducted. In 12 studies (21%) anaesthesia was reported for neither the mother nor the foetus/ neonate. The use of maternal analgesia was reported in only one study. Seventy nine percent of studies (n = 44) did not report the fate of mothers. Twenty percent (n = 11 studies) reported that mothers were killed before or around CS, including 8 that reported manner of death. One study reported that mothers (baboons) were released back to gang cages.

After delivery neonates were either killed or observed for varying lengths of time (up to 6 days). For ventilation studies neonates had endotracheal tubes placed at delivery and were mechanically ventilated for periods ranging from 15 minutes to 24 hours, after which they were killed. Some were given pentobarbital to prevent spontaneous respiration. Some had their tracheas clamped during or after ventilation. Some had catheters inserted, some had agents delivered via intra-tracheal instillations and some had lung fluids aspirated. Foetal / neonatal anaesthesia was reported in 18 studies (32%) but in 25 studies where neonates survived for a period after delivery no anaesthesia was reported, including studies that involved ventilation and placement of catheters and endotracheal tubes. No anaesthesia was reported for foetuses undergoing placement of catheters in utero. Four studies (7%) reported using a paralytic agent in neonates, including 2 for which no anaesthesia was reported. No neonatal analgesia was reported.

Neonates/ foetuses were either killed in utero, at delivery, at various time points post-delivery (5 minutes—6 hours), or after various periods of ventilation (15 minutes—24 hours) or observation (1 to 6 days post-delivery). In 4 studies (7%) neonates were observed to see how long it took them to die post-delivery. Ten studies (18%) did not report how neonates were killed and four (7%) did not report when they were killed. Twenty studies (36%) reported deaths before endpoint, including abortions, dead or macerated foetuses in utero, stillbirths and early postnatal deaths. Fifteen studies (27%) reported at least one unexpected event, including pneumothoraces (involving at least 38 neonates), upper airway leaks during tracheal tube placement (7 neonates), pulmonary interstitial emphysema, oedematous foetuses, premature delivery and mothers not pregnant. No studies provided welfare information about neonates. Fifteen studies (27%) reported information about maternal welfare (animal handling, housing, access to food and water, mating, transportation, stress), including one that gave information on post-delivery care. Thirty eight studies (68%) made no ethical statement.

For both mothers and neonates the models were mostly scored as moderate to severe.

#### Thrombolytics for stroke

There were 97 studies of thrombolytics (tissue plasminogen activator, tPA) involving an estimated 6614 animals (3484 rats, 2701 rabbits, 246 mice, 120 guinea pigs, 33 squirrel monkeys, 30 baboons), with an average 68 animals per study. (Two studies did not report numbers so the actual number is higher.) This model involved inducing a stroke and testing the effect of a thrombolytic agent on recovery. On the day before the experiment some animals underwent preparatory surgery under anaesthesia to fabricate a clot, or to place catheters, ligatures or probes. On the day of surgery animals were anaesthetised and a stroke was induced by blocking the carotid and/or cerebral arteries with clots or filaments, or by tying the arteries. During surgery some animals had their skulls opened for the placement of probes and monitors. They were given the thrombolytic agent intravenously. In a variation of the model (11 studies) animals had surgery under anaesthesia to place a catheter, then after recovering from anaesthesia a clot was injected through the catheter to induce a stroke whilst the animals were conscious (and restrained). Anaesthesia was reported in 73 studies (75%). In 13 studies (13%) anaesthesia was reported for some procedures / animal groups but not all and in 11 studies (11%) no anaesthesia was reported. The use of paralytic agents was reported in 6 studies, five in which animals were anaesthetised and one in which baboons had a stroke induced whilst awake. Analgesia was reported in one study.

Post-surgical observation periods could be relatively short (2–24 hours), or could last up to 2 weeks or 2 months. Post-stroke animals frequently had reduced levels of spontaneous activity, rapid involuntary movements of the eye, inability to stand, severely uncoordinated movements and hemiparesis. Some had neurological assessments over 1–4 hours, 1–2 days, or for up to 1 week. Assessments commonly involved being held upside down by the tail or being pushed laterally to test resistance. Others included determining how long rats could remain on a horizontal suspended rotating rod, or how long it took them to remove sticky tape from their paws. Some animals had angiograms, CT or MRI scans post-stroke. For MRI scans animals might be restrained in a head holder with bars in their ears. Anaesthesia appeared to be maintained for scans but this was not always clear. MRI scans could continue for up to 8 hours post stroke, or could be performed at 1, 2 or 7 days.

Sixty two studies (64%) reported how animals were killed and 90% (n = 87) reported when they were killed. Endpoints ranged from up to 6 hours to 2 months, with the most common endpoint being 24 hours post stroke, reported in 35 studies (36%). Nineteen studies (20%) reported endpoints of one week or longer. Thirty seven studies (38%) reported deaths prior to endpoint, mainly in the first 24 hours post-stroke. The causes included haemorrhage, cerebral oedema and ‘technical reasons’. Seventeen studies (18%) reported some type of unexpected event including technical difficulties, failure of procedures, haemorrhage, major bleeding complications, secondary stroke and fatal hypotension. Only 18 studies (19%) reported information on welfare (housing/ temperature and light / access to food and water/ animal purchase / monitoring of experiments / depth of anaesthesia), including one study that noted attempts to attenuate suffering. None gave details of post-operative care. Sixty seven studies (69%) made an ethical statement.

Most scorers categorised the stroke models as severe. Some commented that if the study duration was brief and anaesthesia/ analgesia were used, then studies might score less severely. Scorers categorised models that induced stroke while animals were conscious as severe.

#### Manner of death (all interventions)

Sixty eight percent of studies (n = 145) reported how animals were killed. The most frequently used method was an overdose of anaesthesia but a wide variety of additional methods were employed. In 7 studies (6 of which were for antenatal corticosteroids) animals were left to die. Methods of death for foetuses included tracheal blockage, strangulation and suffocation.

#### Summary of harms

Most of the studies involved severe or mostly severe harms to animals, as indicated both by the data extracted on procedures and the severity classification by the expert panel. As the extracted data support the severity classifications (Table 6) we have used the latter when conducting the HBA.

The only studies that involved mostly moderate harms were those on bisphosphonates. A greater percentage of bisphosphonate studies reported welfare information than other interventions and these studies also reported relatively few deaths before endpoint. Bisphosphonate studies may have involved the least harms for animals.

### Clinical relevance

As noted above, clinical relevance is indicated by concordance between the findings of animal and human studies for the same treatment intervention, *and* current clinical practice. Antifibrinoloytics reduce surgical bleeding and the need for transfusion in humans and are widely used in current clinical practice;[25] however the animal studies produced inconclusive data.[22] Bisphosphonates increase bone mineral density in animals and in post-menopausal women with osteoporosis.[22] They are recommended as a primary preventative treatment for post-menopausal women with osteoporosis.[26] Corticosteroids benefit animals with head injury but increase the risk of mortality in humans.[22] They are no longer recommended for routine use in people with traumatic head injury.[27] Tirilazad reduces infarct volume and improves neuro-behavioural scores in animals but increases the risk of death and dependency in humans.[22] It is considered to have no role in the current treatment of stroke.[28] Antenatal corticosteroids reduce respiratory distress in both animal and human neonates. They also reduce mortality in humans but the mortality data were inconclusive in animals.[22] The routine clinical use of antenatal corticosteroids for preterm delivery is recommended in hospital settings in high income countries.[29] Thrombolytics reduce infarct volume and improve neuro-behavioural scores in animals but increase haemorrhage risk; in humans they reduce death or dependency despite an increase in haemorrhage.[22] Thrombolytics are used to treat certain categories of ischaemic stroke as long as patients present up to 4.5 hours post-stroke (ideally within 3 hours) and relevant expertise and infrastructure is available,[30, 31] however there has been some controversy over their use.[32–34]

Concordance appears to relate to clinical use, with fully and partially concordant treatment interventions being in clinical use (see Table 7). This suggests that concordance is a good indicator of clinical relevance. Fully concordant interventions (bisphosphonates, thrombolytics) also used relatively low numbers of animals (Table 6) and a greater percentage of studies in fully concordant interventions also made ethical statements (Table 3) and had pharmaceutical company involvement (Table 1) than other interventions. Studies for these interventions were also conducted more recently (Table 1).

**Table 7:** Clinical relevance.

	Concordance	In clinical use
**Antifibrinolytics**	**Discordance**: animal data inconclusive but outcomes improved in humans	**Yes**: widely used in clinical practice
**Corticosteroids**	**Discordance**: improve outcomes in animals but increase mortality in humans	**No**: no longer routinely recommended for traumatic head injury
**Tirilazad**	**Discordance**: improves outcomes in animals but increases mortality in humans	**No**: considered to have no role in the treatment of stroke
**Antenatal corticosteroids**	**Partial concordance**: reduces respiratory distress in both animals and humans; reduces mortality in humans but animal mortality data inconclusive	**Yes**: routine use in hospitals in high income countries is recommended
**Thrombolysis**	**Concordance**: improves outcomes in animals and humans but with risk of haemorrhage	**Yes**: recommended for use with certain stroke patients if they present within 4.5 hours post stroke and if relevant expertise is available. Use has been considered controversial.
**Bisphosphonates**	**Concordance**: improves outcomes in animals and humans	**Yes**: recommended as primary preventative treatment for post-menopausal women with osteoporosis

### Research importance

As noted above, research importance refers to the quality of the research as well as its broader impact (indicated here by citation scores). Perel et al rated the quality of research as ‘poor’ for studies across all 6 interventions. The average citation scores for the studies ranged from 10 for antifibrinolytics to 76 for bisphosphonates (Table 8), with 50 being the average for all studies.

**Table 8:** Citation scores.

Intervention	No. studies	Total no. citations	Average citation score	Research quality
Antifibrinolytics	8	83	10	Poor
Bisphosphonates	16	1213	76	Poor
Corticosteroids	17	610	36	Poor
Tirilizad	18	820	45	Poor
Antenatal corticosteroids	56	2483	44	Poor
Thrombolytics	97	5370	55	Poor

The citation scores appeared to track clinical relevance, with the two highest scores being for the fully concordant interventions, bisphosphonates and thrombolytics (Table 9).

**Table 9:** Citation scores related to clinical relevance.

	Concordance	In clinical use	Average citation score
**Antifibrinolytics**	Discordant	Yes	10
**Corticosteroids**	Discordant:	No	36
**Tirilazad**	Discordant:	No	45
**Antenatal corticosteroids**	Partially concordant:	Yes	44
**Thrombolysis**	Concordant:	Yes	55
**Bisphosphonates**	Concordant:	Yes	76

### Retrospective harm-benefit analysis

**Fig 2:**
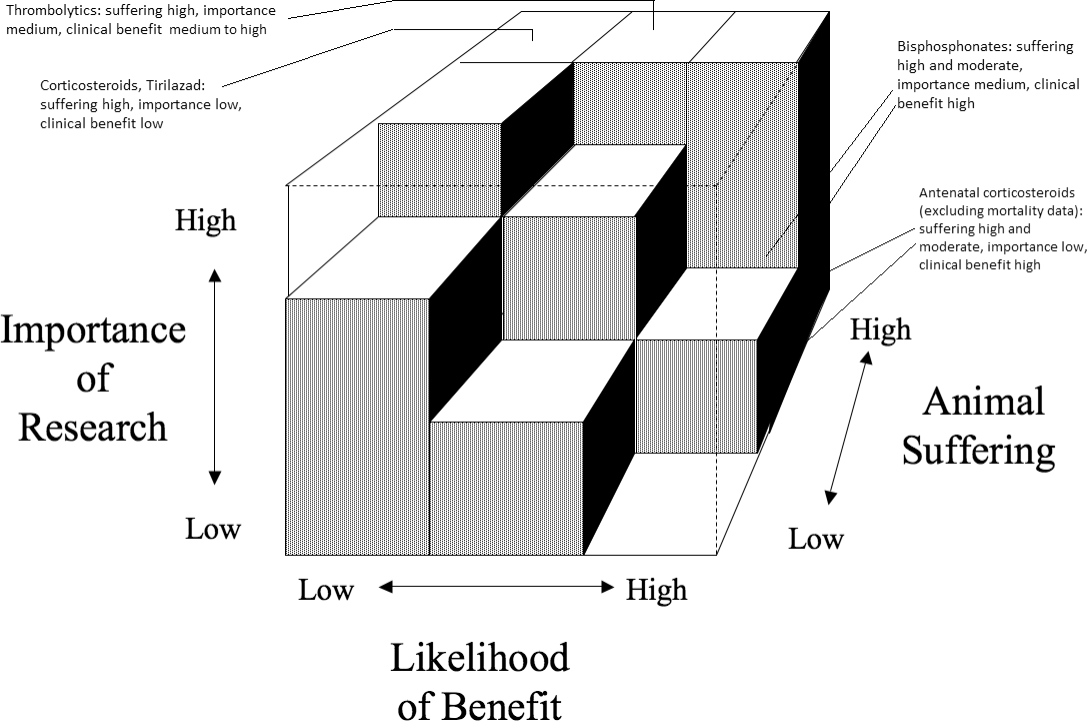
HBA using Bateson’s cube.

As noted above, if the three assessments (animal suffering, likelihood of benefit, research importance) fall into the solid part of Bateson’s Cube, the project is deemed unacceptable. We performed the HBA by conducting the three assessments for each of the six treatment interventions (Fig 2).

For the studies of corticosteroids and Tirilazad there was high animal suffering and low (no) clinical benefit. Their research importance is considered to be low since the quality of research was poor and their citation scores were below average. As such these studies fall into the solid part of the cube and should not have been approved. The thrombolytic studies involved high animal suffering. Their research importance is considered to be medium since although the research quality was poor, their citation scores were above average. Due to controversy regarding the use of thrombolytics, as well as the conditions placed upon their use, their clinical benefit is considered to be medium to high. Because of the high animal suffering these studies fall into the solid part of the cube and should not have been approved. The bisphosphonate studies involved mostly moderate, but also some severe, harms. As such they are considered to involve medium and high animal suffering. The research importance for the bisphosphonate studies is considered to be medium because although the citation scores were well above average, the quality of research studies was poor. The clinical benefit of these studies is considered to be high. As such the studies involving moderate suffering fall into the clear part of the cube and were permissible, while those involving severe suffering fall into the solid part and were not permissible.

It was not possible to assess concordance where animal studies had produced inconclusive data, and as concordance was a necessary component of clinical relevance, it made the HBA impossible. Consequently, it was not possible to conduct a HBA for antifibrinolytics, as the data from the animal studies were inconclusive. However the fact that the animal data were inconclusive, combined with the studies’ poor research quality and very low citation scores, suggests that the clinical use of antifibrinolytics may have developed independently of the animal studies. In the case of antenatal corticosteroids, mortality data from the animal studies were inconclusive but there was concordance between the animal and human studies in terms of reducing neonatal respiratory distress. The antenatal corticosteroid studies involved mostly moderate to severe harms for both mothers and neonates. As such they are considered to involve high and medium animal suffering. The research importance for the antenatal corticosteroid studies is considered to be low because their research quality was poor and citation scores are below average. Consequently, despite the clinical benefit of these studies being considered high (not counting the inconclusive mortality data), they fall into the solid parts of the cube and were not permissible.

The moderate bisphosphonate studies then, are the only studies that fall into the clear part of the cube in that they were of medium research importance, appeared to minimise harms to animals and were associated with benefits for humans.

## Discussion

### Study limitations

Despite expert help we were unable to find 15 of the original studies, all of which reported research on thrombolytics. However, as the thrombolytic studies were by far the most numerous and because we located the majority of them (n = 97), we are confident that the missing papers did not bias our findings. Furthermore, the thrombolytic studies were homogenous in terms of models used so the missing papers were unlikely to have presented any unusual findings.

This is a re-analysis of an existing study so any limitations in the original study design are reproduced here. Although the sample of interventions was not random it provided a range, both in terms of interventions and in the spread of concordance, discordance and partial concordance. As such it provides a good testing ground for analysing harms and benefits and is appropriate for the purposes of this study. The methodological quality of the animal studies for all 6 interventions was poor; this is not unusual for animal studies[35–39] but it may explain why some of the animal data were inconclusive.

It is possible that the severity classifications could reflect poor reporting of measures taken to alleviate suffering. Members of the scoring panel noted that in the absence of information about analgesia, or duration of anaesthesia, they had to classify some procedures as severe that may potentially have scored less severely. However, it is likely that the classifications reflect actual harms since evidence suggests, for example, that if pain relief is not reported then it was not administered.[40] The classification system itself is not very discriminating and although other systems exist for classifying harms,[41] this one was chosen because it is used by EU regulatory bodies and is similar to the American system.[23]

In terms of exploring concordance between animal and human studies it is important to note that many variables may influence the outcome of both animal and human studies, including research design and reporting and publication bias. Perel et al, for example, found strong evidence of publication bias in their systematic review of animal studies of thrombolytics which may have resulted in the treatment effects of thrombolytics being overestimated. Consequently there is a need for caution when interpreting concordance. Furthermore, concordance does not imply causation, i.e. while it may provide an indication of clinical relevance, it does not necessarily imply that the animal studies led directly to human benefit. Indeed the publication dates of the studies suggest that clinical trials did not follow on directly from animal studies; the animal and human studies often appeared to run concurrently and in some cases human studies preceded animal studies, while in others animal studies continued to be published after the treatment effect was known in humans (see Table 2). This confusing picture suggests a lack of communication between those conducting animal research and those running clinical trials.[22]

### Feasibility

We have confirmed the feasibility, both of conducting a retrospective HBA and of collecting data on animal harms from pre-clinical research publications. We found Bateson’s cube to be helpful in guiding the HBA as the principle is clear, namely that research falling into the solid parts of the cube should not be approved. As such it follows a strict principle of disallowing research that is of poor quality or that causes severe suffering.

### Harms

The studies involved an estimated 27,149 animals, including non-human primates. The most common assessment of animal harms by the expert panel was ‘severe’. Reported use of analgesia was rare and some animals (including most neonates) endured significant procedures with no, or only light, anaesthesia reported. Some animals suffered iatrogenic harms. Many were kept alive for long periods post-experimentally but only 1% of studies reported post-operative care. A third of studies reported that some animals died prior to endpoints.[1] Directive 2010/63/EU (Annex IV) specifies acceptable and unacceptable methods for killing different species of animals, indicating that some of the ways animals in these studies were killed would no longer be considered acceptable.[1] Severe harms however, continue to be permitted within current regulatory frameworks; both Directive 2010/63/EU[1] and current US policy[23] allow severe unalleviated pain, suffering or distress, although it requires strong justification. EU requirements in 2014 to record *actual*, as well as predicted, harms[42] and to retrospectively assess individual projects categorised as severe may eventually lead to greater adoption of refinement measures, but there is still clearly an urgent need to review regulations that permit animals to suffer severe harms. In the UK the ASC has recently recommended that every establishment and ethical review board develops ways to avoid procedures involving severe suffering, with the ultimate goal of eliminating severe suffering altogether.[12]

Given that many of the studies are now several years old it might be argued that the sort of harms reported in these studies would no longer be inflicted on animals, but critically, we found no indication of a trend towards improvement; thrombolytic studies, of which more were conducted in the 2000s than in other decades (mode year of publication 2002) and corticosteroid studies (mode year of publication 2005), continued to inflict severe harms on animals. All of the studies reviewed here were conducted prior to the publication in 2010 and 2011 of the ARRIVE Guidelines[43] and the Gold Standard Publication Checklist[44] for reporting animal research. As these guidelines are intended to improve not only reporting but also research quality and animal welfare the hope might be that they will ultimately reduce animal suffering. However, neither of these guidelines require reporting on the use of restraints and paralytics, the fate of mothers where foetuses or neonates are used, details of additional related procedures, the severity classification of the research nor humane endpoints (although the ARRIVE guidelines require reporting of welfare-related assessments). Furthermore, the ARRIVE guidelines do not yet appear to have resulted in improved reporting standards.[45] Similarly, while more of the journals that publish animal experiments now require author assurance of adherence to ethical standards,[46] compliance with ethical guidelines does not necessarily lead to actual improvements in animal welfare,[47] nor does nor the fact that experiments have gained ethical approval.[48, 49] Guidelines have the potential to improve reporting standards, provide clear data[50] and ultimately improve animal welfare, but they require enforcement from all concerned, including journals and reviewers.

Our study suggests that bodies involved in funding, reviewing and authorising animal studies need to pay greater attention to possibilities for refinement, particularly the use of predefined humane endpoints and the consistent use of anaesthesia and analgesia, including for neonates and foetuses. Had such refinements been employed in these studies they may well have reduced animal suffering. There is increasing attention to the refinement component of the 3Rs[40, 47, 48, 51] as well as its potential impact on research[52] and this is to be welcomed. However, given the increasing range of non-animal technologies available[53] the potential for replacement should also be robustly addressed at the stage that projects are being considered for approval.

### Retrospective HBA

This was not an ‘audit’ of the prospective HBA process; an HBA was not a legal requirement for most of these studies since they were either conducted in countries that do not require a formal HBA, or in Europe prior to 2013. Nevertheless, this is the first time a systematic retrospective HBA of a range of pre-clinical animal studies has been conducted and it is perhaps remarkable that the fitness for purpose and accountability of the prospective HBA has not been previously investigated in this way. As noted above, public support for animal research is conditional upon the minimisation of harms to animals and upon benefits to humans and other animals. This HBA found that that the majority of studies involved severe animal suffering. Many animals suffered severe harms that were not associated with human benefit. Only the moderate bisphosphonate studies, less than 7% of the total, appeared to minimise harms to animals whilst being associated with benefit for humans. Some studies (corticosteroids and Tirilazad) not only inflicted severe suffering on animals but were associated with increased human mortality. The regulatory systems in place when these studies were conducted failed to safeguard animals from severe suffering or to ensure that only beneficial, scientifically rigorous research was conducted.

At present responsibility for the prospective authorisation of animal studies is spread amongst research teams, ethical review boards (Animal Welfare and Ethical Review Boards, or AWERBs in the UK), peer reviewers of funding applications, and licensing bodies (such as the UK’s Home Office).[54] Consequently, crucial checks relating to scientific rigour, likelihood of benefit, the availability of non-animal technologies and the assessment and minimisation of animal harms may be conducted in an inconsistent and disparate manner, with no guarantee that the bodies involved hold the appropriate expertise. Research quality for example, is—in the UK at least–mainly the responsibility of peer reviewers who assess funding applications to determine whether the sample size and experimental design are appropriate.[54] However, the poor quality of the animal studies reviewed here and elsewhere[35, 37, 39, 55, 56] suggests that these aspects are not adequately addressed in the peer review process. To ensure that the HBA is fit for purpose, project applications involving animals need to be rigorously scrutinised to ensure that only scientifically robust studies that minimise animal harms and have a strong likelihood of benefit are authorised. This requires both more accurate prospective assessments and tighter regulatory procedures.

This retrospective HBAs highlights factors that may improve the prospective assessment of animal studies. Whilst research quality and animal harms are already assessed prospectively, it is difficult to predict in advance how important or beneficial a research study will be. Our study found that higher citation scores for animal studies appeared to relate to their clinical relevance. This raises the possibility of using citation scores to investigate a research team’s track record, permitting insight into the likely impact of their future studies. Accurate prediction of a study’s future benefits is more challenging, however, and relatively little effort has gone into improving the accuracy of this aspect of the prospective HBA.[2] The problem is compounded by a tendency among scientists to be over optimistic about the potential benefits of their research, particularly when seeking funding.[57, 58] This optimism, together with an implicit confidence in animal research,[59, 60] is likely to bias assessments towards a prediction of benefit. Yet increasing doubts about the validity of findings derived from animal studies[35–39, 55, 61–65] and their translation to humans[20, 22, 66–70] suggest that such confidence may be unwarranted. So how can the likelihood of only authorising beneficial pre-clinical animal research be increased? First, we found that concordance between animal and human studies appeared to relate to clinical relevance. If animal and human studies within the field of interest are ongoing and are being conducted concurrently (as is often the case), concordance between the animal and human data in that field can be assessed as part of a prospective HBA to investigate the likelihood of the animal studies having clinical relevance. Second, our research found that a greater percentage of studies in concordant interventions had pharmaceutical company involvement; it is possible that pharmaceutical companies influence experimental design for the better, or involve greater communication between pre-clinical and clinical scientists, leading to greater relevance. Further research is needed to explore whether this is the case and what the implications might be.

In terms of regulation, ethical review boards have been described as the lynchpin of the HBA[12] and we suggest that the robustness of the HBA might be enhanced by increasing the level, range and consistency of expertise on such boards so that, in addition to considering animal welfare and refinements, each board includes an expert in statistics and experimental design to guarantee scientific rigour (and ensure that animals’ lives are not ‘wasted’) and an expert in non-animal technologies to guarantee that animal use is absolutely necessary. To enhance the likelihood of only beneficial pre-clinical animal studies being authorised, we recommend that funding and licensing bodies make it a requirement for project applicants, as part of their application, to conduct a systematic review of animal studies in their field[71, 72] and to relate the systematic review findings to the relevant clinical research. This would allow reviewers to assess the status and strength of the evidence in the field, the need for the proposed study (thus avoiding unnecessary replication) and the extent of concordance with any existing clinical data. We also suggest that funding bodies, licensing bodies, peer reviewers and ethical review boards adopt a precautionary approach when assessing animal studies so that instead of automatically making an assumption of benefit, they in fact presume (in line with the evidence noted above) that the research is unlikely to be beneficial. These measures would place the burden of proof on those submitting proposals and encourage them to make a much stronger case for any anticipated benefits. Finally, the involvement of experts from a greater range of disciplines (e.g. epidemiology, public health, clinical research) in the reviewing and authorisation of studies would enable wider questions to be asked, such as ‘Is the aim of this project appropriate?’ and if so, ‘Is animal research the best way to answer it?’

In terms of research, we recommend that the relevance to humans of pre-clinical animal research is systematically evaluated,[73] an undertaking that the UK government’s chief scientific advisor also regards as important. In his 2016 lecture to the animal research community, he asked: ‘To what extent have we as a community, ever subjected our claims about how vital animal research has been to human health to the same level of scrutiny we’d apply to those claiming to have discovered a new cure? And I think if not, we must.’[74]

## Conclusion

The HBA is a cornerstone of animal research regulation and is considered to be a key ethical safeguard for animals. This is the first time its accountability has been systematically explored across a range of pre-clinical animal studies. This HBA found that that the majority of studies involved severe animal suffering. Many animals suffered severe harms that were not associated with benefits for humans. Only a small proportion of studies minimised harms to animals whilst being associated with human benefit. The regulatory systems in place when these studies were conducted failed to safeguard animals from severe suffering or to ensure that only beneficial, scientifically rigorous research was conducted. Our findings indicate an urgent need to: i. review regulations, particularly those that permit animals to suffer severe harms; ii. reform the processes of prospectively assessing pre-clinical animal studies to make them fit for purpose; and iii. systematically evaluate the relevance to humans of bodies of pre-clinical animal research that have already been conducted, to provide a more realistic assessment of its likely future benefits and increase the accuracy of prospective HBAs.

## Supporting information

S1 File References to the 228 animal studies. (DOCX)

S1 Table Additional procedures involving further animals. (DOCX)

S2 Table Results of expert panel severity classifications. (DOCX)
