# Supplementary material for: Retrospective harm benefit analysis of pre-clinical animal research for six treatment interventions
Source: PLoS One. 2018 Mar 28;13(3):e0193758. doi: 10.1371/journal.pone.0193758 (PMC5874012; doi:10.1371/journal.pone.0193758)
Supplement: S1 File — (DOCX) [file pone.0193758.s001.docx]

**S1 File: References to the 228 animal studies**

**References that were retrieved (n = 212)**

***Corticosteroids***

1. Yamaguchi M, Shirakata S, Taomoto K, Matsumoto S. Steroid treatment of brain edema. Surg Neurol 1975;4(1):5-8.

2. Itani AL, Ducker TB. Effect of high and low doses of steroids on head injuries. Surg Forum 1976;27(62):478-80.

3. Tornheim PA, McLaurin RL. Effect of dexamethasone on cerebral edema from cranial impact in the cat. J Neurosurg 1978;48(2):220-7.

4. Hausdoerfer J, Heller W. Metabolic events in acutely damaged brain and the effect of dexamethasone in guinea pigs. Resuscitation 1979;7(2):101-7.

5. Rivas JM, Rivas A, Mateos JH. Effects of dexamethasone on experimental lesions of rat brain. Arch Invest Med (Mex) 1983;14(2):153-8.

6. Hall ED. High-dose glucocorticoid treatment improves neurological recovery in head-injured mice. J Neurosurg 1985;62(6):882-7.

7. Hall ED, McCall JM, Chase RL, Yonkers PA, Braughler JM. A nonglucocorticoid steroid analog of methylprednisolone duplicates its high-dose pharmacology in models of central nervous system trauma and neuronal membrane damage. J Pharmacol Exp Ther 1987;242(1):137-42.

8. Shapira Y, Davidson E, Weidenfeld Y, Cotev S, Shohami E. Dexamethasone and indomethacin do not affect brain edema following head injury in rats. J Cereb Blood Flow Metab 1988;8(3):395-402.

9. Hall ED, Yonkers PA. Comparison of two ester prodrugs of methylprednisolone on early neurologic recovery in a murine closed head injury model. J Neurotrauma 1989;6(3):163-8.

10. Spillert CR, Glicini RL, Tortella BJ, Lazaro EJ. Effect of steroid therapy in experimental head trauma. Brain Inj 1990;4(2):199-201.

11. Shapira Y, Artru AA, Yadid G, Shohami E. Methylprednisolone does not decrease eicosanoid concentrations or edema in brain tissue or improve neurologic outcome after head trauma in rats. Anesth Analg 1992;75(2):238-44.

12. Ildan F, Polat S, Oner A, et al. The effect of the treatment of high-dose methylprednisolone on Na(+)-K(+)/Mg(+2) ATPase activity and lipid peroxidation and ultrastructural findings following cerebral contusion in rat. Surg Neurol 1995;44(6):573-80.

13. Holmin S, Mathiesen T. Dexamethasone and colchicine reduce inflammation and delayed oedema following experimental brain contusion. Acta Neurochir (Wien) 1996;138(4):418-24.

14. Park CO. The effects of methylprednisolone on prevention of brain edema after experimental moderate diffuse brain injury in rats--comparison between dosage, injection time, and treatment methods. Yonsei Med J 1998;39(5):395-403.

15. Koc RK, Kurtsoy A, Pasaoglu H, Karakucuk EI, Oktem IS, Meral M. Lipid peroxidation and oedema in experimental brain injury: comparison of treatment with methylprednisolone, tirilazad mesylate and vitamin E. Res Exp Med (Berl) 1999;199(1):21-8.

16. Yang JT, Lee TH, Weng HH, et al. Dexamethasone enhances NT-3 expression in rat hippocampus after traumatic brain injury. Exp Neurol 2005;192(2):437-43.

17. Gahm C, Holmin S, Rudehill S, Mathiesen T. Neuronal degeneration and iNOS expression in experimental brain contusion following treatment with colchicine, dexamethasone, tirilazad mesylate and nimodipine. Acta Neurochir (Wien) 2005;147(10):1071-84.

***Antifibrinolytics***

18. De Nicola P, Luscher EF, Soardi F, Gibelli A, Cipolli PL. Modifications of bleeding time and intensity of prothrombin consumption and of fibrinolysis in rats after administration of platelet extracts alone and with antifibrinolytic agents. *Farmaco [Sci]* 1967;22(8):627-40.

19. Laungani GB, Beyer MM, Friedman EA. Epsilon-aminocaproic acid in traumatic renal hematuria in the rabbit. *Invest Urol* 1975;12(6):458-60.

20. Stenberg B, Karlsson L, Alpsten M, Risberg B. Reduction of gastric haemorrhage by fibrinolysis inhibition - an experimental study in rats. *Thromb Haemost* 1983;49(2):106-8.

21. Berstad A, Almodovar K, Berstad K, Anderson W, Hirschowitz BI. Effect of tranexamic acid on gastric bleeding in rats. *Scand J Gastroenterol* 1988;23(4):402-6.

22. Arnljots B, Wieslander JB, Dougan P, Salemark L. Importance of fibrinolysis in limiting thrombus formation following severe microarterial trauma: an experimental study in the rabbit. *Microsurgery* 1991;12(5):332-9.

23. Racanelli AL, Diemer MJ, Dobies AC, Dubin JR, Reilly TM. Comparison of recombinant plasminogen activator inhibitor-1 and epsilon amino caproic acid in a hemorrhagic rabbit model. *Thromb Haemost* 1992;67(6):692-6.

24. Samama CM, Mazoyer E, Bruneval P, et al. Aprotinin could promote arterial thrombosis in pigs: a prospective randomized, blind study. *Thromb Haemost* 1994;71(5):663-9.

25. Thomae KR, Mason DL, Rock WA, Jr. Randomized blinded study of aprotinin infusion for liver crush injuries in the pig model. *Am Surg* 1997;63(2):113-20.

***Thrombolysis***

26. Kissel P, Chehrazi B, Seibert JA et al. Digital angiographic quantification of blood flow dynamics in embolic stroke treated with tissue-type plasminogen activator. J Neurosurg 1987; 67:399-405

27. Penar PL, Greer CA. The effect of intavenous tissue-type plasminogen activator in a rat model of embolic cerebral ischemia. Yale Journal of Biology and Medicine. 1987; 60:233-244

28. Slivka A, Pulsinelli W. Hemorrhagic complications of thrombolytic therapy in experimental stroke. Stroke. 1987; 18:1148-1156

30. Phillips DA, Fisher M, Smith TW et al. The safety and angiographic efficacy of tissue plasminogen activator in a cerebral embolization model. Annals of Neurology. 1988; 23:391-394

31. Zivin JA, Lyden PD, Degirolami U et al. Tissue plasminogen activator. Reduction of neurologic damage after experimental embolic stroke. Archives of Neurology. 1988; 45:387-391

32. Chehrazi B, Seibert JA, Kissel P et al. Evaluation of recombinant tissue plasminogen activator in embolic stroke. Neurosurgery. 1989; 24:355-360

33. del Zoppo GJ, Copeland BR, Anderchek K et al. Hemorrhagic transformation following tissue plasminogen activator in experimental cerebral infarction. Stroke. 1990; 21:596-601

34. Lyden PD, Zivin JA, Clark WA et al. Tissue plasminogen activator-mediated thrombolysis of cerebral emboli and its effect on hemorrhagic infarction in rabbits. Neurology. 1989; 39:703-708

35. Phillips DA, Fisher M, Smith TW et al. The effects of a new tissue plasminogen activator analogue, Fb-Fb-CF, on cerebral reperfusion in a rabbit embolic stroke model. Annals of Neurology. 1989; 25:281-285

37. Bednar MM, McAuliffe T, Raymond S et al. Tissue plasminogen activator reduces brain injury in a rabbit model of thromboembolic stroke. Stroke. 1990; 21:1705-1709

38. Benes V, Zabramski JM, Boston M et al. Effect of intra-arterial tissue plasminogen activator and urokinase on autologous arterial emboli in the cerebral circulation of rabbits [corrected]. Stroke. 1990; 21:1594-1599

39. Hara T, Iwamoto M, Ogawa H et al. Dissolution of emboli in rats with experimental cerebral thromboembolism by recombinant human tissue plasminogen activator (TD-2061). Thrombosis Research. 1990; 59:703-712

40. Lyden PD, Madden K, Clark WM et al. Incidence of cerebral hemorrhage after treatment with tissue plasminogen activator or streptokinase following embolic stroke in rabbits [corrected]. Stroke. 1990; 21:1589-1593

41. Phillips DA, Fisher M, Davis MA et al. Delayed treatment with a t-PA analogue and streptokinase in a rabbit embolic stroke model. Stroke. 1990; 21:602-605

42. Clark WM, Madden K, Lyden PD et al. Cerebral hemorrhagic risk of aspirin or heparin therapy with thrombolytic treatment in rabbits. Stroke. 1991; 22:872-876

43. Carter LP, Guthkelch AN, Orozco J et al. Influence of tissue plasminogen activator and heparin on cerebral ischemia in a rabbit model. Stroke. 1992; 23:883-888

44. Matsuno H, Uematsu T, Umemura K et al. A simple and reproducible cerebral thrombosis model in rat induced by a photochemical reaction and the effect of a plasminogen-plasminogen activator chimera in this model. Journal of Pharmacological &Toxicological Methods. 1993; 29:165-173

45. Thomas GR, Thibodeaux H, Bennett WF et al. Optimized thrombolysis of cerebral clots with tissue-type plasminogen activator in a rabbit model of embolic stroke. Journal of Pharmacology and Experimental Therapeutics. 1993; 264:67-73

46. Umemura K, Wada K, Uematsu T et al. Evaluation of the combination of a tissue type plasminogen activator, SUN9216, and a thromboxane A2 receptor antagonist, vapiprost, in a rat middle cerebral artery thrombosis model. Stroke. 1993; 24:1077-1082

47. Hamilton MG, Lee JS, Cummings PJ et al. A comparison of intra-arterial and intravenous tissue-type plasminogen activator on autologous arterial emboli in the cerebral circulation of rabbits. Stroke. 1994; 25:651-656

48. Sakurama T, Kitamura R, Kaneko M. Tissue-type plasminogen activator improves neurological functions in a rat model of thromboembolic stroke. Stroke. 1994; 25:451-456

49. Thomas GR, Thibodeaux H, Errett CJ et al. A long-half-life and fibrin-specific form of tissue plasminogen activator in rabbit models of embolic stroke and peripheral bleeding. Stroke. 1994; 25:2072-2078

50. Umemura K, Toshima Y, Nakashima M. Thrombolytic efficacy of a modified tissue type plasminogen activator, SUN9216, in the rat middle cerebral artery thrombosis model. European Journal of Pharmacology. 1994; 262:27-31

51. Gross CE, Raymond SJ, Howard DB et al. Delayed tissue-plasminogen activator therapy in a rabbit model of thromboembolic stroke. Neurosurgery. 1995; 36:1172-1177

52. Orozco J, Mendel RC, Hahn MR et al. Influence of a 'brain protector' drug 21-amino steroid on the effects of experimental embolic stroke treated by thrombolysis. Neurological Research. 1995; 17:423-425

53. Bowes MP, Zivin JA, Thomas GR et al. Acute hypertension, but not thrombolysis, increases the incidence and severity of hemorrhagic transformation following experimental stroke in rabbits. Experimental Neurology. 1996;40-46

54. Lekieffre D, Benavides J, Scatton B et al. Neuroprotection afforded by a combination of eliprodil and a thrombolytic agent, rt-PA, in a rat thromboembolic stroke model. Brain Research. 1997; 76:88-95

55. Vanderschueren S, Van Vlaenderen I, Collen D. Intravenous thrombolysis with recombinant staphylokinase versus tissue-type plasminogen activator in a rabbit embolic stroke model. Stroke. 1997; 28:1783-1788

56. Yenari MA, De Crespigny A, Palmer JT et al. Improved perfusion with rt-PA and hirulog in a rabbit model of embolic stroke. Journal of Cerebral Blood Flow and Metabolism. 1997; 17:401-411

58. Busch E, Krueger K, Allegrini PR et al. Reperfusion after thrombolytic therapy of embolic stroke in the rat: Magnetic resonance and biochemical imaging. Journal of Cerebral Blood Flow and Metabolism. 1998; 18:407-418

59. Hoffman P, Pottier P, Sainte Marie M et al. Clopidogrel enhancement of rt-PA thrombolysis in a thrombo-embolic model of cerebral ischemia in rats. Fibrinolysis and Proteolysis. 1998; 12:97-105

62. Wang YF, Tsirka SE, Strickland S et al. Tissue plasminogen activator (tPA) increases neuronal damage after focal cerebral ischemia in wild-type and tPA-deficient mice. Nature Medicine. 1998; 4:228-231

63. Zhang RL, Chopp M, Zhang ZG et al. Early (1 h) administration of tissue plasminogen activator reduces infarct volume without increasing hemorrhagic transformation after focal cerebral embolization in rats. Journal of the Neurological Sciences. 1998; 160:1-8

64. Brinker G, Franke C, Hoehn M et al. Thrombolysis of cerebral clot embolism in rat: Effect of treatment delay. Neuroreport. 1999;3269-3272

66. Fagan SC, Garcia JH. Hemorrhagic transformation in focal cerebral ischemia: Influence of time to artery reopening and tissue plasminogen activator. Pharmacotherapy. 1999; 19:139-142

67. Hoffmann P, Sainte MM, Bernat A et al. Stroke outcome determination with a prefabricated fibrin-rich macroclot in a thromboembolic rat middle cerebral artery occlusion model. Fibrinolysis & Proteolysis. 1999; 13:193-201

68. Kilic E, Hermann DM, Hossmann K-A. Recombinant tissue plasminogen activator reduces infarct size after reversible thread occlusion of middle cerebral artery in mice. Neuroreport. 1999; 10:107-111

69. Klein GM, Li H, Sun P et al. Tissue plasminogen activator does not increase neuronal damage in rat models of global and focal ischemia. Neurology. 1999; 52:1381-1384

70. Lew SM, Gross CE, Bednar MM et al. Complement depletion does not reduce brain injury in a rabbit model of thromboembolic stroke. Brain Research Bulletin. 1999; 48:325-331

71. Meng W, Wang X, Asahi M et al. Effects of tissue type plasminogen activator in embolic versus mechanical models of focal cerebral ischemia in rats. Journal of Cerebral Blood Flow and Metabolism. 1999; 19:1316-1321

72. Zhang RL, Zhang ZG, Chopp M. Increased therapeutic efficacy with rt-PA and anti-CD18 antibody treatment of stroke in the rat. Neurology. 1999; 52:273-279

73. Asahi M, Asahi K, Wang X et al. Reduction of tissue plasminogen activator-induced hemorrhage and brain injury by free radical spin trapping after embolic focal cerebral ischemia in rats. Journal of Cerebral Blood Flow and Metabolism. 2000; 20:452-457

74. Bednar MM, Gross CE, Russell SR et al. 16(R)-hydroxyeicosatetraenoic acid, a novel cytochrome P450 product of arachidonic acid, suppresses activation of human polymorphonuclear leukocytes and reduces intracranial pressure in a rabbit model of thromboembolic stroke. Neurosurgery. 2000; 47:1410-1419

75. Busch E, Beaulieu C, de Crespigny A et al. MRI demonstrates that tissue-type plasminogen activator increases stroke volume if cerebral arteries are not successfully recanalized.l. Keio University Symposia for Life Science and Medicine. 2000; 6:420-427

77. Jiang Q, Zhang RL, Zhang ZG et al. Magnetic resonance imaging indexes of therapeutic efficacy of recombinant tissue plasminogen activator treatment of rat at 1 and 4 hours after embolic stroke. Journal of Cerebral Blood Flow and Metabolism. 2000; 20:21-27

78. Kilic E, Hermann DM, Hossmann KA. Recombinant tissue-plasminogen activator induced thrombolysis after cerebral thromboembolism in mice. Acta Neuropathologica. 2000; 99:219-222

79. Lapchak PA, Chapman DF, Zivin JA. Metalloproteinase inhibition reduces thrombolytic (tissue plasminogen activator)-induced hemorrhage after thromboembolic stroke. Stroke. 2000; 31:3034-3040

80. Zhang L, Zhang ZG, Chopp M et al. Effects of PS-519 combined with thrombolytic therapy in embolic stroke in rat. Abstracts of the International Stroke Conference. 2000; 32:351-35a

81. Zhang RL, Zhang L, Jiang Q et al. Postischemic intracarotid treatment with TNKtPA reduces infarct volume and improves neurological deficits in embolic stroke in the unanesthetized rat. Brain Res. 2000; 878:64-71

82. Chapman DF, Lyden P, Lapchak PA et al. Comparison of TNK with wild-type tissue plasminogen activator in a rabbit embolic stroke model. Stroke. 2001; 32:748-752

83. Dijkhuizen RM, Asahi M, Wu O et al. Delayed rt-PA treatment in a rat embolic stroke model: Diagnosis and prognosis of ischemic injury and hemorrhagic transformation with magnetic resonance imaging. Journal of Cerebral Blood Flow & Metabolism. 2001; 21:964-971

84. Kilic E, Baehr M, Hermann DM. Effects of recombinant tissue plasminogen activator after intraluminal thread occlusion in mice: Role of hemodynamic alterations. Stroke. 2001; 32:2641-2647

85. Lapchak PA, Chapman DF, Zivin JA. Pharmacological effects of the spin trap agents N-t-butyl-phenylnitrone (PBN) and 2,2,6,6-tetramethylpiperidine-N-oxyl (TEMPO) in a rabbit thromboembolic stroke model: Combination studies with the thrombolytic tissue plasminogen activator. Stroke. 2001; 32:147-153

86. Morris DC, Zhang L, Zhang ZG et al. Extension of the therapeutic window for recombinant tissue plasminogen activator with argatroban in a rat model of embolic stroke. Stroke. 2001; 32:2635-2640

87. Sumii T, Singhal AB, Asahi M et al. Protective effects of pamiteplase, a modified t-PA, in a rat model of embolic stroke. Neuroreport. 2001; 12:615-618

89. Tejima E, Katayama Y, Suzuki Y et al. Hemorrhagic transformation after fibrinolysis with tissue plasminogen activator: Evaluation of role of hypertension with rat thromboembolic stroke model. Stroke. 2001; 32:1336-1340

91. Zhang L, Zhang ZG, Zhang RL et al. Postischemic (6-hour) treatment with recombinant human tissue plasminogen activator and proteasome inhibitor PS-519 reduces infarction in a rat model of embolic focal cerebral ischemia. Stroke. 2001; 32:2926-2931

92. Zhang R, Zhang L, Goussev A et al. Delayed (6h) Intracarotid Treatment with TNKtPA Improves Neurological Functional Recovery after Embolic Stroke in the Unanesthetized Rat. Abstracts of the International Stroke Conference. 2001; 32:352-35e

93. Dijkhuizen RM, Asahi M, Wu O et al. Rapid breakdown of microvascular barriers and subsequent hemorrhagic transformation after delayed recombinant tissue plasminogen activator treatment in a rat embolic stroke model. Stroke. 2002; 33:2100-2104

94. Erdogan B, Sen O, Caner H et al. Intravenous and local intra-arterial tissue plasminogen activator in a rabbit model of acute thromboembolic stroke: Angiographic comparison. Advances in Therapy. 2002; 19:266-274

95. Jiang Q, Zhang RL, Zhang ZG et al. Magnetic resonance imaging characterization of hemorrhagic transformation of embolic stroke in the rat. Journal of Cerebral Blood Flow and Metabolism. 2002; 22:559-568

96. Maeda M, Furuichi Y, Ueyama N et al. A combined treatment with tacrolimus (FK506) and recombinant tissue plasminogen activator for thrombotic focal cerebral ischemia in rats: increased neuroprotective efficacy and extended therapeutic time window. Journal of Cerebral Blood Flow & Metabolism. 2002; 22:1205-1211

97. Muramatsu H, Igarashi H, Okubo S et al. Monteplase reduces infarct volume and hemorrhagic transformation in rat model of embolic stroke. Neurological Research. 2002; 24:311-316

98. Neumann-Haefelin C, Brinker G, Uhlenkuken U et al. Prediction of Hemorrhagic Transformation After Thrombolytic Therapy of Clot Embolism: An MRI Investigation in Rat Brain. Stroke. 2002; 33:1392-1398

99. Niessen F, Hilger T, Hoehn M et al. Thrombolytic treatment of clot embolism in rat: comparison of intra-arterial and intravenous application of recombinant tissue plasminogen activator. Stroke. 2002; 33:2999-3005

100. Shuaib A, Yang Y, Nakada MT et al. Glycoprotein IIb/IIIa antagonist, murine 7E3 F(ab') 2, and tissue plasminogen activator in focal ischemia: evaluation of efficacy and risk of hemorrhage with combination therapy. Journal of Cerebral Blood Flow & Metabolism. 2002; 22:215-222

101. Toomey JR, Valocik RE, Koster PF et al. Inhibition of factor IX(a) is protective in a rat model of thromboembolic stroke. Stroke. 2002; 33:578-585

103. Zhang Z, Zhang L, Yepes M et al. Adjuvant treatment with neuroserpin increases the therapeutic window for tissue-type plasminogen activator administration in a rat model of embolic stroke. Circulation. 2002; 106:740-745

104. Gautier S, Petrault O, Gele P et al. Involvement of thrombolysis in recombinant tissue plasminogen activator-induced cerebral hemorrhages and effect on infarct volume and postischemic endothelial function. Stroke. 2003; 34:2975-2979

105. Grobholz K, Burggraf D, Martens HK et al. rt-PA Attenuates Basal Lamina Antigen Loss after Focal Cerebral Ischemia. Stroke. 2003; 34:306

106. Lapchak PA, Zivin JA. Ebselen, a seleno-organic antioxidant, is neuroprotective after embolic strokes in rabbits: Synergism with low-dose tissue plasminogen activator. Stroke. 2003; 34:2013-2018

107. Okubo S, Igarashi H, Yamaguchi H et al. Therapeutic time window of rt-PA on embolic stroke in rat. In: Abe K, ed. Molecular Mechanism and Epochal Therapeutics of Ischemic Stroke and Dementia.: Elsevier, 2003:203-207

108. Suzuki M, Sasamata M, Miyata K. Neuroprotective effects of YM872 coadministered with t-PA in a rat embolic stroke model. Brain Research. 2003; 959:169-172

109. Suzuki Y, Kano T, Katayama Y et al. Reduction of infarction volume by bolus injection of pamiteplase, a modified tissue plasminogen activator with a longer half-life. Neurological Research. 2003; 25:477-480

110. Yang Y, Li Q, Yang T et al. Reduced brain infarct volume and improved neurological outcome by inhibition of the NR2B subunit of NMDA receptors by using CP101,606-27 alone and in combination with rt-PA in a thromboembolic stroke model in rats. Journal of Neurosurgery. 2003; 98:397-403

112. Daffertshofer M, Huang Z, Fatar M et al. Efficacy of sonothrombolysis in a rat model of embolic ischemic stroke. Neuroscience Letters. 2004; 361:115-119

113. Ding G, Jiang Q, Zhang L et al. Multiparametric ISODATA analysis of embolic stroke and rt-PA intervention in rat. Journal of the Neurological Sciences. 2004; 223:135-143

114. Kollmar R, Henninger N, Bardutzky J et al. Combination therapy of moderate hypothermia and thrombolysis in experimental thromboembolic stroke--an MRI study. Experimental Neurology. 2004; 190:204-212

115. Lapchak PA, Song D, Wei J et al. Pharmacology of caffeinol in embolized rabbits: clinical rating scores and intracerebral hemorrhage incidence. Experimental Neurology. 2004; 188:286-291

116. Moriguchi A, Mihara K, Aoki T et al. Restoration of middle cerebral artery thrombosis by novel glycoprotein IIb/IIIa antagonist FK419 in guinea pig. European Journal of Pharmacology. 2004; 498:179-188

117. Suzuki Y, Nagai N, Collen D. Comparative effects of microplasmin and tissue-type plasminogen activator (tPA) on cerebral hemorrhage in a middle cerebral artery occlusion model in mice. Journal of Thrombosis & Haemostasis Vol 2(9)()(pp 1617-1621), 2004. 2004;1617-1621

119. Yi JS, Kim YH, Koh JY. Infarct reduction in rats following intraventricular administration of either tissue plasminogen activator (tPA) or its non-protease mutant S478A-tPA. Experimental Neurology. 2004; 189:354-360

120. Zhang W, Sato K, Hayashi T et al. Extension of ischemic therapeutic time window by a free radical scavenger, Edaravone, reperfused with tPA in rat brain. Neurological Research. 2004; 26:342-348

121. Ding G, Jiang Q, Zhang L et al. Analysis of combined treatment of embolic stroke in rat with r-tPA and a GPIIb/IIIa inhibitor. Journal of Cerebral Blood Flow & Metabolism. 2005; 25:87-97

123. Kilic E, Kilic U, Matter CM et al. Aggravation of focal cerebral ischemia by tissue plasminogen activator is reversed by 3-hydroxy-3-methylglutaryl coenzyme A reductase inhibitor but does not depend on endothelial NO synthase. Stroke. 2005; 36:332-336

124. Maeda M, Moriguchi A, Mihara K et al. FK419, a nonpeptide platelet glycoprotein IIb/IIIa antagonist, ameliorates brain infarction associated with thrombotic focal cerebral ischemia in monkeys: Comparison with tissue plasminogen activator. Journal of Cerebral Blood Flow & Metabolism. 2005; 25:108-118

125. Busch E, Krueger K, Hossmann KA. Improved model of thromboembolic stroke and rt-PA induced reperfusion in the rat. Brain Research. 1997; 778:16-24

126. Busch E, Kruger K, Fritze K et al. Blood-Brain barrier disturbances after rt-PA treatment of thromboembolic stroke in the rat. Acta Neurochir Suppl. 1997; 70:206-208

127. Jiang Q, Zhang ZG, Zhang RL et al. Diffusion- and perfusion-weighted NMR imaging study of middle cerebral artery thrombotic focal ischemia and rt-PA intervention in rat. Fibrinolysis & Proteolysis. 1998; 12:33-43

128. Jiang Q, Zhang RL, Zhang ZG et al. Diffusion-, T2-, and perfusion-weighted nuclear magnetic resonance imaging of middle cerebral artery embolic stroke and recombinant tissue plasminogen activator intervention in the rat. Journal of Cerebral Blood Flow and Metabolism. 1998; 18:758-767

130. Zhang RL, Zhang ZG, Chopp M. Thrombolysis with tissue plasminogen activator alters adhesion molecule expression in the ischemic rat brain. Stroke. 1999; 30:624-629

131. Kano T, Katayama Y, Tejima E et al. Hemorrhagic transformation after fibrinolytic therapy with tissue plasminogen activator in a rat thromboembolic model of stroke. Brain Research. 2000; 854:245-248

133. Lapchak PA, Araujo DM, Song D et al. The nonpeptide glycoprotein IIb/IIIa platelet receptor antagonist SM-20302 reduces tissue plasminogen activator-induced intracerebral hemorrhage after thromboembolic stroke. Stroke. 2002; 33:147-152

134. Lapchak PA, Araujo DM, Song D et al. Effects of the spin trap agent disodium-[tert-butylimino)methyl]benzene-1,3-disulfonate N-oxide (generic NXY-059) on intracerebral hemorrhage in a rabbit Large clot embolic stroke model: combination studies with tissue plasminogen activator. Stroke. 2002; 33:1665-1670

135. Zhang L, Zhang ZG, Zhang R et al. Adjuvant treatment with a glycoprotein IIb/IIIa receptor inhibitor increases the therapeutic window for low-dose tissue plasminogen activator administration in a rat model of embolic stroke. Circulation. 2003; 107:2837-2843

136. Zhang L, Zhang ZG, Zhang RL et al. Effects of a selective CD11b/CD18 antagonist and recombinant human tissue plasminogen activator treatment alone and in combination in a rat embolic model of stroke. Stroke. 2003; 34:1790-1795

137. Zhang L, Zheng GZ, Zhang C et al. Intravenous administration of a GPIIb/IIIa receptor antagonist extends the therapeutic window of intra-arterial tenecteplase-tissue plasminogen activator in a rat stroke model. Stroke. 2004; 35:2890-2895

***Tirilazad***

139. Lythgoe, D. J., Little, R. A., O'Shaughnessy, C. T., and Steward, M. C. Effect of U- 74006-F on edema and infarct volumes following permanent occlusion of the middle cerebral artery in the rat. Br.J.Pharmacol. 100, 454P. 1990.

140. Beck T, Bielenberg GW. The effects of two 21-aminosteroids on overt infarct size 48 hours after middle cerebral artery occlusion in the rat. Brain Res 1991;560:159-62.

141. Xue D, Slivka A, Buchan AM. Tirilazad reduces cortical infarction after transient but not permanent focal cerebral ischemia in rats. Stroke 1992;23:894-99.

142. Wilson JT, Bednar MM, McAuliffe TL, Raymond S, Gross CE. The effect of the 21-aminosteroid U74006F in a rabbit model of thromboembolic stroke. Neurosurgery 1992;31:929-33.

143. Takeshima R, Kirsch JR, Koehler RC, Traystman RJ. Tirilazad treatment does not decrease early brain injury after transient focal ischemia in cats. Stroke 1994;25:670-676.

144. Hellstrom HO, Wanhainen A, Valtysson J, Persson L, Hillered L. Effect of tirilazad mesylate given after permanent middle cerebral artery occlusion in rat. Acta Neurochir (Wien ) 1994;129:188-92.

145. Park CK, Hall ED. Dose-response analysis of the effect of 21-aminosteroid tirilazad mesylate (U-74006F) upon neurological outcome and ischemic brain damage in permanent focal cerebral ischemia. Brain Res 1994;645:157-63.

146. Umemura K, Wada K, Uematsu T, Mizuno A, Nakashima M. Effect of 21-aminosteroid lipid peroxidation inhibitor, U74006F, in the rat middle cerebral artery occlusion model. Eur J Pharmacol 1994;251:69-74.

147. Orozco J, Mendel RC, Hahn MR, Guthkelch AN, Carter LP. Influence of a 'brain protector' drug 21-amino steroid on the effects of experimental embolic stroke treated by thrombolysis. Neurol Res 1995;17:423-25.

148. Gross CE, Kimelberg HK, Raymond-Russell S, Booth C, Bednar MM. Delayed adjuvant therapy with the 21-aminosteroid U74006F and the anion channel blocker L644-711 does not improve outcome following thrombolytic therapy in a rabbit model of thromboembolic stroke. Surg Neurol 1997;47:60-65.

149. Schmid-Elsaesser R, Zausinger S, Hungerhuber E, Baethmann A, Reulen HJ. Monotherapy with dextromethorphan or tirilazad--but not a combination of both improves outcome after transient focal cerebral ischemia in rats. Exp Brain Res 1998;122:121-27.

150. Alessandri B, Basciani R, Langemann H et al. Chronic effects of an aminosteroid on microdialytically measured parameters after experimental middle cerebral artery occlusion in the rat. J Clin Neurosci 2000;7:47-51.

151. Oktem IS, Menku A, Akdemir H, Kontas O, Kurtsoy A, Koc RK. Therapeutic effect of tirilazad mesylate (U-74006F), mannitol, and their combination on experimental ischemia. Res Exp Med (Berl) 2000;199:231-42.

152. Scholler K, Zausinger S, Baethmann A, Schmid-Elsaesser R. Neuroprotection in ischemic stroke--combination drug therapy and mild hypothermia in a rat model of permanent focal cerebral ischemia. Brain Res 2004;1023:272

153. Schmid-Elsaesser R, Hungerhuber E, Zausinger S, Baethmann A, Reulen HJ. Combination drug therapy and mild hypothermia: a promising treatment strategy for reversible, focal cerebral ischemia. Stroke 1999;30:1891-99.

154. Schmid-Elsaesser R, Hungerhuber E, Zausinger S, Baethmann A, Reulen HJ. Neuroprotective efficacy of combination therapy with two different antioxidants in rats subjected to transient focal ischemia. Brain Research 1999;816:471-79.

155. Zausinger S, Schoeller K, Plesnila N, Schmid-Elsaesser R. Combination drug therapy and mild hypothermia after transient focal cerebral ischemia in rats. Stroke 2003;34:2246-51.

156. Zausinger S, Westermaier T, Plesnila N, Steiger HJ, Schmid-Elsaesser R. Neuroprotection in transient focal cerebral ischemia by combination drug therapy and mild hypothermia: comparison with customary therapeutic regimen. Stroke 2003;34:1526-32.

***Antenatal corticosteroids***

157. Asoh K, Kumai T, Murano K, Kobayashi S, Koitabashi Y. Effect of antenatal dexamethasone treatment on Ca2+-dependent nitric oxide synthase activity in rat lung. *Pediatric Research.* 2000;48:91-5.

158. Ballard PL, Ning Y, Polk D, Ikegami M, Jobe AH. Glucocorticoid regulation of surfactant components in immature lambs. *American Journal of Physiology.* 1997;273:1048-57.

159. Barrada MI, Blomquist CH, Kotts C. The effects of betamethasone on fetal development in the rabbit. *Am.J.Obstet.Gynecol.* 1980;136:234-8.

160. Beck JC, Mitzner W, Johnson JWC. Betamethasone and the rhesus fetus: Effect on lung morphometry and connective tissue. *Pediatric Research.* 1981;15:235-40.

161. Bunt JEH, Carnielli VP, Seidner SR, Ikegami M, Wattimena JLD, Sauer PJJ *et al*. Metabolism of endogenous surfactant in premature baboons and effect of prenatal corticosteroids. *American Journal of Respiratory & Critical Care Medicine.* 1999;160:1481-5.

162. Devaskar U, Church JC, Chechani V, Sadiq F. Effect of simultaneous administration of betamethasone and triiodothyronine (T3) on the development of functional pulmonary maturation in fetal rabbit. *Biochemical & Biophysical Research Communications.* 1987;146:524-9.

163. ElKady T,.Jobe A. Maternal treatments with corticosteroids and/or T3 change lung volumes and rupture pressures in preterm rabbits. *Biology of the Neonate.* 1988;54:203-10.

164. Engle MJ, Kemnitz JW, Rao TJ, Perelman RH, Farrell PM. Effects of maternal dexamethasone therapy on fetal lung development in the rhesus monkey. *American Journal of Perinatology. 13(7):399-407,* 1996.

165. Fiascone JM, Jacobs HC, Moya FR, Mercurio MR, Lima DM. Betamethasone increases pulmonary compliance in part by surfactant-independent mechanisms in preterm rabbits. *Pediatric Research.* 1987;22:730-5.

166. Gladstone IM, Mercurio MR, Devenny SG, Jacobs HC. Antenatal steroids, postnatal surfactant, and pulmonary function in premature rabbits. *Journal of Applied Physiology.* 1989;67:1377-82.

167. Ikegami M, Berry D, ElKady T, Pettenazzo A, Seidner S, Jobe A. Corticosteroids and surfactant change lung function and protein leaks in the lungs of ventilated premature rabbits. *J.Clin.Invest* 1987;79:1371-8.

168. Ikegami M, Jobe AH, Seidner S, Yamada T. Gestational effects of corticosteroids and surfactant in ventilated rabbits. *Pediatric Research.Vol.25(1)()(pp 32-37), 1989.*1989;32-7.

169. Ikegami M, Jobe A, Pettenazzo A, Seidner S. Effect of maternal hormone treatment on lung protein leakage and lung function of preterm newborn rabbits. *European Respiratory Journal* 1989;Supplement.:16s-20s.

170. Ikegami M, Jobe AH, Newnham J, Polk DH, Willet KE, Sly P. Repetitive prenatal glucocorticoids improve lung function and decrease growth in preterm lambs. *American Journal of Respiratory & Critical Care Medicine.* 1997;156:178-84.

171. Jobe AH, Newnham J, Willet K, Sly P, Ikegami M. Fetal versus maternal and gestational age effects of repetitive antenatal glucocorticoids. *Pediatrics.* 1998;102:1116-25.

172. Johnsson H, Eriksson L, Sedin G. Antenatal betamethasone administration decreases the lung hyaluronan concentration in preterm rabbit pups. *Pediatric Research.* 2001;49:566-71.

173. Kessler DL, Truog WE, Murphy JH. Experimental hyaline membrane disease in the premature monkey. Effects of antenatal dexamethasone. *American Review of Respiratory Disease.* 1982;126:62-9.

174. Mitzner W, Johnson JWC, Scott R. Effect of betamethasone on pressure-volume relationship of fetal rhesus monkey lung. *Journal of Applied Physiology.* 1979;47:377-82.

175. Morga FA, Riquelme RA, Lopez AA, Moya FR, Llanos AJ. Maternal administration of glucocorticoid and thyrotropin-releasing hormone enhances fetal lung maturation in undisturbed preterm lambs. *American Journal of Obstetrics & Gynecology.* 1994;171:729-34.

176. Motoyama EK, Orzalesi MM, Kikkawa Y, Kaibara M, Wu B, Zigas CJ *et al*. Effect of cortisol on the maturation of fetal rabbit lungs. *Pediatrics* 1971;48:547-55.

177. Pillow JJ, Hall GL, Willet KE, Jobe AH, Hantos Z, Sly PD. Effects of gestation and antenatal steroid on airway and tissue mechanics in newborn lambs. *Am.J.Respir.Crit Care Med.* 2001;163:1158-63.

178. Pratt L, Magness RR, Phernetton T, Hendricks SK, Abbott DH, Bird IM. Repeated use of betamethasone in rabbits: Effects of treatment variation on adrenal suppression, pulmonary maturation, and pregnancy outcome. *American Journal of Obstetrics & Gynecology.* 1999;180:995-1005.

179. Rider ED, Jobe AH, Ikegami M, Yamada T, Seinder S. Antenatal betamethasone dose effects in preterm rabbits studied at 27 days gestation. *Journal of Applied Physiology.* 1990;68:1134-41.

180. Rooney SA, Gobran LI, Chu AJ. Thyroid hormone opposes some glucocorticoid effects on glycogen content and lipid synthesis in developing fetal rat lung. *Pediatric Research.* 1986;20:545-50.

181. Sun B, Jobe A, Rider E, Ikegami M. Single dose versus two doses of betamethasone for lung maturation in preterm rabbits. *Pediatr.Res.* 1993;33:256-60.

182. Tabor BL, Ikegami M, Jobe AH, Yamada T, Oetomo SB. Dose response of thyrotropin-releasing hormone on pulmonary maturation in corticosteroid-treated preterm rabbits. *American Journal of Obstetrics & Gynecology.* 1990;163:669-76.

183. Tan RC, Ikegami M, Jobe AH, Li YY, Possmayer F, Ballard PL. Developmental and glucocorticoid regulation of surfactant protein mRNAs in preterm lambs. *American Journal of Physiology - Lung Cellular & Molecular Physiology.* 1999;277:L1142-L1148.

184. Tsao FH, Gutcher GR, Zachman RD. Effect of hydrocortisone on the metabolism of phosphatidylcholine in maternal and fetal rabbit lungs and livers. *Pediatr.Res.* 1979;13:997-1001.

185. Ward JA, Erenberg A, Roberts RJ. Postnatal increase in airway surfactant in the premature rabbit exposed in utero to betamethasone. *Developmental Pharmacology & Therapeutics.* 1983;6:388-403.

186. Willet KE, Jobe AH, Ikegami M, Kovar J, Sly PD. Lung morphometry after repetitive antenatal glucocorticoid treatment in preterm sheep. *American Journal of Respiratory & Critical Care Medicine.* 2001;163:1437-43.

187. Zaremba W, Grunert E, Aurich JE. Prophylaxis of respiratory distress syndrome in premature calves by administration of dexamethasone or a prostaglandin F-2 alpha analogue to their dams before parturition. *American Journal of Veterinary Research* 1997;58:404-7.

188. Moss TJ, Mulrooney NP, Nitsos I, Ikegami M, Jobe AH, Newnham JP. Intraamniotic corticosteroids for preterm lung maturation in sheep. *Am.J.Obstet.Gynecol.* 2003;189:1389-95.

189. Anderson GG, Lamden MP, Cidlowski JA, Ashikaga T. Comparative pulmonary surfactant-inducing effect of three corticosteroids in the near-term rat. *American Journal of Obstetrics & Gynecology.* 1981;139:562-4.

190. Frank L, Lewis PL, Sosenko IR. Dexamethasone stimulation of fetal rat lung antioxidant enzyme activity in parallel with surfactant stimulation. *Pediatrics.75(3):569-74,* 1985;75:569-74.

191. Phelps DS,.Floros J. Dexamethasone in vivo raises surfactant protein B mRNA in alveolar and bronchiolar epithelium. *American Journal of Physiology - Lung Cellular & Molecular Physiology.* 1991;260:L146-L152.

192. Shimizu H, Miyamura K, Kuroki Y. Appearance of surfactant proteins, SP-A and SP-B, in developing rat lung and the effects of in vivo dexamethasone treatment. *Biochimica et Biophysica Acta - Lipids & Lipid Metabolism.* 1991;1081:53-60.

193. Lohninger AK, Bock P, Salzer H, Sevelda P, Lohninger AF. Antenatal betamethasone-dose-effects on fetal rat lung morphology and surfactant. *Journal of Perinatal Medicine. Vol.22(4)()(pp 319-328), 1994.* 1994;319-28.

194. Yokoyama N, Takada S, Uetani Y, Nakamura H. Effects of maternal administration of dexamethasone and thyrotropin-releasing hormone on fetal rat pulmonary surfactant synthesis. *Biology of the Neonate.Vol.68(1)()(pp 39-46), 1995.* 1995;39-46.

195. Chen C-M, Ikegami M, Ueda T, Polk DH, Jobe AH. Fetal corticosteroid and T4 treatment effects on lung function of surfactant-treated preterm lambs. *American Journal of Respiratory & Critical Care Medicine.Vol.151(1)()(pp 21-26), 1995.* 1995;21-6.

196. Kikkawa Y, Kaibara M, Motoyama EK, Orzalesi MM, Cook CD. Morphologic development of fetal rabbit lung and its acceleration with cortisol. *Am J Pathol.* 1971;64:423-42.

197. Taeusch HW, Jr., Heitner M, Avery ME. Accelerated lung maturation and increased survival in premature rabbits treated with hydrocortisone. *American Review of Respiratory Disease.* 1972; 105:971-3.

198. Corbet AJ, Flax P, Alston C, Rudolph AJ. Effect of aminophyllin and dexamethasone on secretion of pulmonary surfactant in fetal rabbits. *Pediatr.Res.* 1978;12:797-9.

199. Thibeault DW, Emmanouilides GC, Dodge ME. Pulmonary and circulatory function in preterm lambs treated with hydrocortisone in utero. *Biol.Neonate* 1978;34:238-47.

200. Johnson JWC, Limb HS, Kearney K. The effect of hydrocortisone on lamb lung function. *Journal of Reproductive Medicine for the Obstetrician & Gynecologist.* 1981;26:413-20.

201. Hallmann M, Teramo K, Sipinen S, Raivio K. Effects of betamethasone and ritodrine on the fetal secretion of lung surfactant. *Journal of Perinatal Medicine.* 1985;13:23-9.

202. Warburton D, Parton L, Buckley S, Cosico L, Enns G, Saluna T. Combined effects of corticosteroid, thyroid hormones, and beta-agonist on surfactant, pulmonary mechanics, and beta-receptor binding in fetal lamb lung. *Pediatr.Res.* 1988;24:166-70.

203. Ikegami M, Polk D, Tabor B, Lewis J, Yamada T, Jobe A. Corticosteroid and thyrotropin-releasing hormone effects on preterm sheep lung function. *Journal of Applied Physiology. Vol.70(5)()(pp 2268-2278), 1991.* 1991;2268-78.

204. Jobe AH, Polk D, Ikegami M, Newnham J, Sly P, Kohen R *et al*. Lung responses to ultrasound-guided fetal treatments with corticosteroids in preterm lambs. *Journal of Applied Physiology.Vol.75(5)()(pp 2099-2105), 1993.* 1993;2099-105.

205. Stein HM, Martinez A, Blount L, Oyama K, Padbury JF. The effects of corticosteroids and thyrotropin-releasing hormone on newborn adaptation and sympathoadrenal mechanisms in preterm sheep. *American Journal of Obstetrics & Gynecology.* 1994;171:17-24.

206. Ueda T, Ikegami M, Polk D, Mizuno K, Jobe A. Effects of fetal corticosteroid treatments on postnatal surfactant function in preterm lambs. *Journal of Applied Physiology.* 1995;79:846-51.

207. Ikegami M, Polk D, Jobe A. Minimum interval from fetal betamethasone treatment to postnatal lung responses in preterm lambs. *American Journal of Obstetrics & Gynecology.* 1996;174:1408-13.

208. Rebello CM, Ikegami M, Polk DH, Jobe AH. Postnatal lung responses and surfactant function after fetal or maternal corticosteroid treatment. *Journal of Applied Physiology.* 1996;80:1674-80.

209. Polk DH, Ikegami M, Jobe AH, Sly P, Kohan R, Newnham J. Preterm lung function after retreatment with antenatal betamethasone in preterm lambs. *Am.J.Obstet.Gynecol.* 1997;176:308-15.

210. Rebello CM, Ikegami M, Ervin MG, Polk DH, Jobe AH. Postnatal lung function and protein permeability after fetal or maternal corticosteroids in preterm lambs. *Journal of Applied Physiology.* 1997;84:213-8.

211. Ervin MG, Seidner SR, Leland MM, Ikegami M, Jobe AH. Direct fetal glucocorticoid treatment alters postnatal adaptation in premature newborn baboons. *American Journal of Physiology - Regulatory Integrative & Comparative Physiology.* 1998;274:1169-76.

212. Flecknoe SJ, Boland RE, Wallace MJ, Harding R, Hooper SB. Regulation of alveolar epithelial cell phenotypes in fetal sheep: Roles of cortisol and lung expansion. *American Journal of Physiology - Lung Cellular & Molecular Physiology.* 2004;L1207-L1214.

***Bisphosphonates***

213. Balena R, Toolan BC, Shea M, Markatos A, Myers ER, Lee SC *et al*. The Effects of 2-Year Treatment with the Aminobisphosphonate Alendronate on Bone Metabolism, Bone Histomorphometry, and Bone Strength in Ovariectomized Nonhuman-Primates. *Journal of Clinical Investigation* 1993;92:2577-86.

214. Seedor JG, Quartuccio HA, Thompson DD. The bisphosphonate alendronate (MK-217) inhibits bone loss due to ovariectomy in rats. *Journal of Bone & Mineral Research.* 1991;6:339-46.

215. Thompson DD, Seedor JG, Quartuccio H, Solomon H, Fioravanti C, Davidson J *et al*. The bisphosphonate, alendronate, prevents bone loss in ovariectomized baboons. *Journal of Bone & Mineral Research.7(8):951-60,* 1992.

216. Lauritzen DB, Balena R, Shea M, Seedor JG, Markatos A, Le HM *et al*. Effects of Combined Prostaglandin and Alendronate Treatment on the Histomorphometry and Biomechanical Properties of Bone in Ovariectomized Rats. *Journal of Bone and Mineral Research* 1993;8:871-9.

217. Frolik CA, Bryant HU, Black EC, Magee DE, Chandrasekhar S. Time-dependent changes in biochemical bone markers and serum cholesterol in ovariectomized rats: Effects of raloxifene HCl, tamoxifen, estrogen, and alendronate. *Bone* 1996;18:621-7.

218. Sato M, Bryant HU, Iversen P, Helterbrand J, Smietana F, Bemis K *et al*. Advantages of raloxifene over alendronate or estrogen on nonreproductive and reproductive tissues in the long-term dosing of ovariectomized rats. *Journal of Pharmacology & Experimental Therapeutics.* 1996;279:298-305.

219. Giardino R, Fini M, Aldini NN, Gnudi S, Biagini G, Gandolfi MG *et al*. Salmon synthetic calcitonin and alendronate effects on bone quality in ovariectomized rats. [Italian]. *Minerva Medica.* 1997;88:469-77.

220. Azuma Y, Oue Y, Kanatani H, Ohta T, Kiyoki M, Komoriya K. Effects of continuous alendronate treatment on bone mass and mechanical properties in ovariectomized rats: Comparison with pamidronate and etidronate in growing rats. *Journal of Pharmacology & Experimental Therapeutics.* 1998;286:128-35.

221. Da Paz LHBC, De F, V, Teng NC, Dos Reis LM, Pereira RMR, Jorgetti V. Effect of 17-estradiol or alendronate on the bone densitometry, bone histomorphometry and bone metabolism of ovariectomized rats. *Brazilian Journal of Medical & Biological Research.* 2001;34:1015-22.

222. Giavaresi G, Fini M, Gnudi S, Nicoli AN, Rocca M, Carpi A *et al*. Comparison of calcitonin, alendronate and fluorophosphate effects on ovariectomized rat bone. *Biomedicine & Pharmacotherapy.* 2001;55:397-403.

223. Wang X, Yang Z, Yu S. The treatment of osteoporosis and bone resorption of alveoli with alendronate in rat model. [ChiNESE. *Chung-Hua Kou Chiang i Hsueh Tsa Chih Chinese Journal of Stomatology.36(3):193-6,* 2001.

224. Ito M, Azuma Y, Takagi H, Komoriya K, Ohta T, Kawaguchi H. Curative effect of combined treatment with alendronate and 1a-hydroxyvitamin D<inf>3</inf> on bone loss by ovariectomy in aged rats. *Japanese Journal of Pharmacology.* 2002;89:255-66.

225. Ito M, Azuma Y, Takagi H, Kamimura T, Komoriya K, Ohta T *et al*. Preventive effects of sequential treatment with alendronate and 1 alpha-hydroxyvitamin D-3 on bone mass and strength in ovariectomized rats. *Bone* 2003;33:90-9.

226. Pytlik M, Kaczmarczyk-Sedlak I, Sliwinski L, Janiec W, Rymkiewicz I. Effect of concurrent administration of alendronate sodium and retinol on development of changes in histomorphometric parameters of bones induced by ovariectomy in rats. *Polish Journal of Pharmacology.* 2004;56:571-9.

227. Sliwinski L, Janiec W, Pytlik M, Folwarczna J, Kaczmarezyk-Sedlak I, Pytlik W *et al*. Effect of administration of alendronate sodium and retinol on the mechanical properties of the femur in ovariectomized rats. *Polish Journal of Pharmacology* 2004;56:817-24.

228. Liu ZP, Li WX, Yu B, Huang J, Sun J, Huo JS *et al*. Effects of trans-resveratrol from Polygonum cuspidatum on bone loss using the ovariectomized rat model. *Journal of Medicinal Food.* 2005;8:14-9.

**References that could not be found (n = 15)**

29. Del Zoppo GJ, Copeland BR, Hacke W et al. Intracerebral Hemorrhage following rtPA infusion in a primate stroke model. Stroke. 1988; 19:134

36. Takigami M. Studies on the therapeutic efficacy of fibrinolytic agents in acute experimental thromboembolic stroke use of a new model of cerebral embolism in rabbits using an autologous arterial white thrombus. Sapporo Medical Journal. 1989; 58:25-38

57. Zhang RL, Chopp M, Zhang ZG et al. Tissue Plasminogen Activator Induces Thrombolysis of a Cerebral Thrombus and Reduces Cerebral Infarct Volume in Rat. Journal of Cerebral Blood Flow & Metabolism. 1997; 17:313

60. Huang J, Kim LJ, Kisiel W et al. Inhibition of factor IXa-dependent coagulation improves efficacy of tPA in stroke without increasing intracerebral hemorrhage. Circulation. 1998; 98:I1-1016

61. Ito T, Yoshimura SI, Okumura A et al. The effect of tissue plasminogen activator (tPA) on proximal middle cerebral artery (MCA) thrombosis in hamster. European Journal of Neurology. 1998; 5:S49

76. Hara T, Mies G, Hata R et al. Effect of thrombolysis on the dynamics of ischemic injury after clot embolism of middle cerebral artery in mice. European Journal of Neuroscience. 2000; 12:220

*(NB: A paper by Hara et al (2000) has an almost identical title but as it is unclear whether it reports the same experiment the decision was made not to accept it as a substitute.)*

88. Suzuki Y, Kano T, Katayama Y et al. Reduction of infarction volume by bolus injection of long-acting tissue plasminogen activator. Nihon University Journal of Medicine. 2001; 43:293-300

90. Warren DT, Klein GM, Buchan AM. Enhanced inflammation may be the mechanism of tissue plasminogen activator toxicity in a rat model of permanent focal ischemia. Neurology. 2001; 56:A370

102. Warren D, Barber P, Zhao Z et al. MK 801 Protects Tissue Plasminogen Activator Mediated Neuroexcitotoxicity in Permenant Focal Ischemia. Stroke. 2002; 33:405

111. Back T, Otto D, Kittner D et al. Failure to enhance thrombolytic therapy by neuroprotection with memantine in embolic stroke. Akt Neurol. 2004; 31

118. Uddin G, Shuaib A, Hussain M et al. Adjuvant threatment with Glyco-Protein IIb/IIIa antagonist reduces perfusion deficits and improves cerebral blood circulation after middle cerebral artery occlusion, in rat model of embolic stroke. 5th World Stroke Conference. 2004;

122. Hamman GF, Burggraff D, Liebetrau M et al. TNK Increases Microvascular Integrity Loss Following Focal Cerebral Ischemia and Reperfusion in the Rat. Stroke. 2005; 36:421

129. Zhang R, Jiang Q, Zhang Z et al. Therapeutic window and hemorrhagic transformation after rht-PA treatment of cerebral embolic stroke in rats. Stroke. 1999; 30:246

132. Kano T, Tejima E, Katayama Y. Hemorrhagic transformation after fibrinolysis in a rat thromboembolic model. Journal of Stroke and Cerebrovascular Diseases 2000: 9: 189-90. (*NB: This paper is also cited as Katayama Y, Kano T, Tejima E. Hemorrhagic transformation after fibrinolysis in a rat thromboembolic model. Journal of Stroke and Cerebrovascular Diseases 2000: 9: 189-90.)*

138. Zhang L, Zhang Z, Zhang C et al. Adjuvant Treatment With Statin Increases the Therapeutic Window for Thrombolysis After Embolic Focal Cerebral Ischemia in Rat. Stroke. 2004; 35:275

**Excluded papers (n = 1)**

65**.** Chopp M, Zhang RL, Zhang ZG et al. The clot thickens--thrombolysis and combination therapies. Acta Neurochir. 1999; 73:67-71. (*Excluded for being a review*)
