# Supplementary material for: Retrospective harm benefit analysis of pre-clinical animal research for six treatment interventions
Source: PLoS One. 2018 Mar 28;13(3):e0193758. doi: 10.1371/journal.pone.0193758 (PMC5874012; doi:10.1371/journal.pone.0193758)
Supplement: S1 Table — (DOCX) [file pone.0193758.s002.docx]

**S1 Table. Additional procedures involving further animals**

|  | **Related procedures reported** | **Additional related procedures involving animals additional to experimental animals*** |
| --- | --- | --- |
| **Antifibrinolytics** | 2/8 | Study 20 used blood from donor rat(s). Study 23 used blood obtained from 14 additional rabbits. |
| **Bisphosphonates** | 0/16 | No related procedures reported. |
| **Corticosteroids** | 0/17 | No related procedures reported. |
| **Tirilizad** | 0/18 | No related procedures reported. |
| **Antenatal corticosteroids** | 3/56 | Study 165 obtained pulmonary surfactant from adult rabbits. Study 192 obtained: surfactant from rats, antisera from white New Zealand rabbits and foetal and neonatal lung tissues from litters of pregnant rats. Study 203 obtained serum from rabbits. |
| **Thrombolytics** | 34/97 | 34 studies of thrombolytics used clots obtained from donors’ blood (of which 5 described the procedures for obtaining the clots). |
| **Total 212 studies** | **39 studies** | **41 procedures reported (clots produced 34; blood ‘donated’ 2; surfactant isolated 2; serum recovered 2; lung tissues obtained 1)** |

*Unless stated otherwise neither the numbers of additional animals involved nor the details of the procedures involved were reported
