# Supplementary material for: Retrospective harm benefit analysis of pre-clinical animal research for six treatment interventions
Source: PLoS One. 2018 Mar 28;13(3):e0193758. doi: 10.1371/journal.pone.0193758 (PMC5874012; doi:10.1371/journal.pone.0193758)
Supplement: S2 Table — (DOCX) [file pone.0193758.s003.docx]

**S2 Tables: Results of expert panel severity classifications**

**Severity classification for antifibrinolytic studies**

| **Summary of animal model**  This model induces blood loss by various methods and then tests anti-fibrinolytic agents that aim to increase clotting/ reduce bleeding. Models were varied. | **Severity score (mild, moderate, severe)** | | | | | | |
| --- | --- | --- | --- | --- | --- | --- | --- |
| **Follow up 2–6 h post-operatively** | **Scorer 1** | **Scorer 2** | **Scorer 3** | **Scorer 4** | **Scorer 5** | **Scorer 6** | **Summary of scores** |
| Rats and rabbits had ears cut/ artery trauma inflicted under anaesthesia, plus at least 2hrs post-operative assessment. Unclear if/ when killed. (studies 22,23) | Moderate (shortish durations) | Moderate | Mild | Moderate | Could be moderate if the bleeding is actually limited and animals killed soon (2hrs) | Probably mild (anaesthetised) | Moderate (3)  Moderate with conditions [bleeding limited, early endpoint] (1)  Mild (2) |
| Pigs had livers injured by crushing and /or induced shock under anaesthesia. They were restrained, paralysed and given painkillers. Some died before 4hrs, rest killed at 4h. (study 25) | Severe | Severe | Severe | Severe | Severe given fundamental damage to vital organs, though relatively short time | Mild as pain controlled and short duration | Likely to be non-recovery* |
| Rats housed in metabolic cage pre-operatively. Gastric bleeding induced by acid under anaesthesia, killed at 6 hrs (study 20) | Severe (acid as trauma) | Severe | Moderate | Severe (unless under anaesthesia) | Severe | Moderate as anaesthesia could have worn off | Likely to be non-recovery* |
| **Follow up 24 h post-operatively** |  |  |  |  |  |  |  |
| Pigs had operation under anaesthesia to produce narrowing of the artery. Ear cutting, some without anaesthesia. Assessed up to 24 hrs post-operatively. Unclear if/ when killed. (study 24) | Severe (not enough detail to conclude otherwise) | Moderate | Mild | Moderate (unless non analgesia, then severe) | Could be moderate if bleeding limited | Moderate to Severe | Mild (1)  Moderate (1)  Moderate with conditions [if analgesia 1; if bleeding limited 1] (2)  Moderate/ severe (1)  Severe (1) |
| **Follow-up 5-10 days post-operatively** |  |  |  |  |  |  |  |
| Rats had tails cut under anaesthesia. Post-operative i.v. administration of test agents for 6 days. Unclear if/ when killed. (study 18) | Moderate (tail non terminal) | Severe | Mild | Moderate (assuming small cut) | Could be moderate if bleeding limited | Severe and pain not controlled post op | Mild (1)  Moderate (1)  Moderate with conditions [small cut 1; bleeding limited] (2)  Severe (2) |
| Rabbits had renal injuries inflicted under anaesthesia. Individually housed in a metabolic cage post-operatively. Urine collected daily for 5-10 days. Killed between 5-10 days. (study 19) | Severe (long duration) | Severe | Moderate | Assuming good analgesia then moderate, otherwise severe | Severe | Severe | Severe (4)  Moderate (1)  Moderate if good analgesia, otherwise severe (1) |
| **Overall summary:** Study of shortest duration (2-6h) generally assessed as moderate, with two scoring mild. Study lasting 24 h had a spread of scores from mild, through moderate to severe, two scoring moderate on condition that bleeding was limited and analgesia given. Studies lasting 5-10 days: the first involved tail cutting under anaesthesia and again produced a spread of scores from mild through moderate to severe. The study that involved rabbits having renal injuries inflicted under anaesthesia with 5-10 day follow up was mostly scored as severe. | | | | | | | |

**Non-recovery studies are those where animals have a general anaesthetic before the procedure and are killed before regaining consciousness. Studies clearly stated to be non-recovery were excluded from scoring, but where it was unclear whether studies were non-recovery, they were included and scored.*

**Severity classification for bisphosphonates studies**

| **Summary of animal model** | **Severity score (mild, moderate, severe)** | | | | | | |
| --- | --- | --- | --- | --- | --- | --- | --- |
|  | **Scorer 1** | **Scorer 2** | **Scorer 3** | **Scorer 4** | **Scorer 5** | **Scorer 6** | **Summary of scores** |
| Typically surgery is conducted under anaesthesia to remove the ovaries, or a sham operation is performed. Treatment with bisphosphonates usually starts straight after operations although in three studies it was delayed to allow for recovery. Drugs (or vehicle) are often given to animals by oral gavage. The period of study ranges from 4 weeks to 2 years. Some animals have injections of fluorescent dye prior to death. Animals were baboons (2 studies), rats (14 studies) | Mild | Moderate if 4 weeks, severe if 2 years | Moderate | Severe unless suitable analgesia, in which case Moderate | Moderate (but not sure of the long-term pain effect of lack of ovaries/ osteoporosis) | On average Moderate | Mild (1)  Moderate (2)  Moderate with conditions [if 4 week endpoint 1; if analgesia 1], otherwise severe (2)  Moderate with qualifications (1) |
| **Variations in model** |  |  |  |  |  |  |  |
| Studies with rats range from 4 weeks to 10 months. Typically they conform to the general model given above but in two studies bone mineral density was also measured, under anaesthesia. Metabolic cages were used in three cases in the days prior to death. | Mild |  | Moderate | Severe unless suitable analgesia, in which case Moderate | Moderate | Moderate | Mild (1)  Missing (1)  Moderate (3)  Moderate with conditions [suitable analgesia] otherwise severe (1) |
| 2 of the studies were on baboons, with one lasting a year and the other 2 years. In these studies baboons were x-rayed, had surgery to remove ovaries and were given drugs every 2 weeks under anaesthesia. Every 3 months and under anaesthesia, they had blood samples taken, bone mineral density measurements done and urine taken by sterile puncture. They had 2 IV injections in the weeks prior to death at 2 yrs. We have no information on housing. | Moderate if repeated anaesthesia, Mild if not. | Moderate  (Recovery from anaesthesia?) | Moderate | Severe | Severe – repeated procedure / handling/ anaesthesia | If housing OK Mild, otherwise Moderate | Moderate (2)  Severe (2)  Mild with conditions [housing OK 1; if no repeated anaesthesia 1], otherwise moderate (2) |
| **Variations in endpoints** |  |  |  |  |  |  |  |
| 2 years | Moderate if repeated anaesthesia, Mild if not. | Severe | Moderate | Severe | Severe | Severe if lots of procedures | Severe (3)  Severe if lots of procedures (1)  Moderate (1)  Mild, unless repeated anaesthesia, in which case moderate (1) |
| 10 months - 1 year | Moderate if repeated anaesthesia, Mild if not. | Severe | Moderate | Severe | Moderate/ Severe | On average Moderate | Severe (2)  Moderate (2)  Moderate/ severe (1)  Mild, unless repeated anaesthesia, in which case moderate (1) |
| 3- 6 months | Moderate if repeated anaesthesia, Mild if not. | Severe | Moderate | Severe | Moderate | On average Moderate | Severe (2)  Moderate (3)  Mild, unless repeated anaesthesia, in which case moderate (1) |
| 4-8 weeks | Moderate if repeated anaesthesia, Mild if not. | Moderate | Moderate | Severe (Moderate if analgesia used) | Moderate | On average Moderate | Moderate (4)  Moderate if analgesia used (1)  Mild, unless repeated anaesthesia, in which case moderate (1) |
| **Additional comments from scorers** |  |  |  |  |  | In this case duration might not impact severity – more a case of housing etc. |  |
| **Overall summary:** Most felt the general model was of moderate severity, but rising to the severe category as the endpoints became longer and if no analgesia was used. Scorer 1 consistently scored procedures as less severe than other scorers. | | | | | | | |

**Severity classification for corticosteroid studies**

| **Summary of animal model** | **Severity scoring (mild, moderate, severe)** | | | | | | |
| --- | --- | --- | --- | --- | --- | --- | --- |
|  | **Scorer 1** | **Scorer 2** | **Scorer 3** | **Scorer 4** | **Scorer 5** | **Scorer 6** | **Summary of scores** |
| This model involves inflicting brain injury and then testing the effect of various corticosteroids on the animal after brain injury. Brain injury was caused in different ways. In one study a hole was drilled in the brain and rubber was put in the brain to apply pressure, later withdrawn and the scalp sutured. In another animals had their heads shaved and were attached to a sled that crashed at speed. In one the scalp was retracted and head injury inflicted with a stunner, requiring intubation for post-injury respiration. One study achieved blood clotting under surgery rather than brain injury. Many had injuries inflicted with devices designed to allow weights to drop on animals’ heads, or with protruding rods. Some had steel caps fitted to their skulls under surgery, prior to head injury. Some had holes drilled in their skulls prior to injury. Some had the scalps sutured after injury.  Post injury medication could by i.v. or i.p. *Not one study reported use of painkillers,* even those that did not use anaesthesia/ light anaesthesia. None reported post-operative care but in studies where animals died before the experimental endpoint it may well have been poor. Details of housing were not given, even for those that were observed over days and weeks. Food and water post-operatively was mentioned in only 2 studies.  Animals were rats (10 studies), mice (4 studies), monkeys (1 study), cats (1 study), guinea pigs (1 study)  Manner of death (where reported): Between 10 – 18% of animals were reported to die upon impact. Some animals were reported to die of their injuries before the experimental endpoint. | Severe (no anaesthesia and no painkillers makes severe) | Severe if animals were conscious or allowed to recover while it was known that they were suffering. | Severe (depends on anaesthesia) | Severe | Severe (especially if uncontrolled damage to skull; non damage to skull more difficult to judge: mild/ severe headache resulting?)  Severe impairment of the animal’s condition seems likely in most cases. | Severe | Severe (3)  Severe if no anaesthesia (3) |
| **Variations of model** |  |  |  |  |  |  |  |
| In three studies mice were *not anaesthetised* (6,7,9). Some of these mice died immediately following injury. The survivors were given test agents and had their neurological status and grip strength assessed after the injury using a string test, whereby the mice were observed to see how long they could stay on the string before falling off onto a table (sometimes padded, sometimes not). We are not told how long the survivors were kept alive for. | Severe (no anaesthesia) | Severe (see above) | Severe | Severe | Severe | Severe | Severe (6) |
| In two studies rats and mice were only *lightly anaesthetised* (8, 10). In one of these studies (10) many animals died within five minutes of injury and most were dead by four days later. Only five survived until the study endpoint which was Day 7. | Severe (despite anaesthesia due to high mortality) | Severe | Severe | Severe | Severe | Severe | Severe (6) |
| ***Endpoints*** Please categorise the severity for each time period: |  |  |  |  |  |  |  |
| up to 2h | Severe | Severe | Severe | Severe unless anaesthetised | Depends on how long the animal is conscious | Severe | Severe (4)  Severe unless anaesthetised (2) |
| up to 1 day | Severe | Severe | Severe | Severe | Severe | Severe | Severe (6) |
| up to 2 days | Severe | Severe | Severe | Severe | Severe | Severe | Severe (6) |
| up to 7 days | Severe | Severe | Severe | Severe | Severe | Severe | Severe (6) |
| up to 14 days | Severe | Severe | Severe | Severe | Severe | Moderate* | Severe (5)  Moderate (1) |
| up to 30 days | Severe | Severe | Severe | Severe | Severe | Moderate* | Severe (5)  Moderate (1) |
| **Additional comments from scorers** |  |  | All severe if cumulative suffering |  |  | *Assume if able to survive this long pain and suffering might have been less? Insufficient info to decide. |  |
| **Overall summary** In general this model was scored as severe. In cases where animals were not anaesthetised or only lightly anaesthetised all scorers categorised procedures as severe. Endpoints were from 2h up to 30 days. Most endpoints were categorised as severe except for studies of the shortest duration which two scorers categorised as moderate as long as anaesthesia was used. For studies lasting 2-4 weeks all but one scorer categorised harms as severe. | | | | | | | |

**Severity classification for tirilazad studies**

| **Summary of animal model** | **Severity scoring (mild, moderate, severe)** | | | | | | |
| --- | --- | --- | --- | --- | --- | --- | --- |
|  | **Scorer 1** | **Scorer 2** | **Scorer 3** | **Scorer 4** | **Scorer 5** | **Scorer 6** | **Summary of scores** |
| The core model is an operation under anaesthesia to occlude the middle cerebral artery (MCAO), or sham, and then to test the effect of Tirilazad on recovery. During the operation probes and monitors may also be placed in the brain or on the surface of the brain. In some studies hypothermia is also induced in animals, presumably under anaesthesia (though this is not always clear). After the operation animals are given test agents or vehicle at various time points, usually intra-peritoneally or intravenously. Some of these test agents may be administered before or during the operation, most are administered post-operatively up to various time points (3h, 4h, 5h, 12 h or 24h).  Post-operatively some animals had MRI scans (24h after operation, anaesthesia usually reported, 2 studies). Neurological assessments include attempting to arouse the rat using tactile and painful stimulation, pushing rat to assess resistance to push and being pulled by tail.  Animals were rats (13 studies), rabbits (4 studies), cats 1 (study) | Severe  (profoundly disabling and long term probably non recoverable injury) | Severe (because they were allowed to recover) | Could be Moderate. Most would be Severe | Severe (unless they are all not conscious as a result of the stroke) | Severe (I guess if the stroke had no obvious effect on suffering (conscious experience) per se, this might change, but there is likely to be ‘severe impairment’ of condition anyway, to warrant Severe | Severe | Severe (6) |
| **Different categories of model and endpoints** |  |  |  |  |  |  |  |
| Animals that had MCAO operation, test agents and were *killed up to 24 hours later*. This category tended to involve more surgical procedures. One study involved a subsequent operation and three involved procedures to produce autologous clots. | Severe (due to 2 procedures) | Severe | Severe | Severe | Severe | Severe | Severe (6) |
| Animals that had MCAO operation, test agents, daily assessments and were *killed up to 2- 3 days later*. A small number of these animals died before the endpoint as a result of haemorrhage due to insertion of monofilament to occlude the MCA. | Severe | Severe | Severe | Severe | Severe | Severe | Severe (6) |
| Animals that had MCAO operation, test agents, daily assessments and *killed at 7 days*. A significant number of these animals died before the endpoint as a result of haemorrhage due to insertion of a monofilament to occlude the MCA. (reported in 7 studies) | Severe (due to significant mortality) | Severe | Severe | Severe | Severe | Severe | Severe (6) |
| **Variations** |  |  |  |  |  |  |  |
| ***Pre-operative additions***  Some studies reported that prior to experiments animals were fasted overnight but allowed free access to water  In 3 studies procedures conducted to produce autologous clots prior to main operation | Pre-op is mild but Severe in total | Severe | Severe | Severe | Severe | Severe | Severe (6) |
| ***Peri-operative variations:*** Paralysis and analgesia were induced with pancuronium bromide and fentanyl in one study | Severe (paralysis requires justification) | Severe | Severe | Severe | Severe | Severe | Severe (6) |
| ***Second operation:*** After an unspecified recovery period a second operation was performed under anaesthesia (to restore flow in the brain) in one study | Severe (2 procedures) | Severe | Severe | Severe | Severe | Severe (less) | Severe (6) |
| **Post-operative care:** Post-operative analgesia reported in one study | If short Severe, if long Moderate | Severe? | Severe | Severe | Severe (but see earlier comment perhaps could be moderate) | Severe (less) | Severe (3)  Potentially moderate/ less severe (3) |
| **Overall summary** The overall model was categorised as severe. Variations of the model were all categorised as severe, except for one study that reported post-operative analgesia; this was categorised as generally severe still, but potentially as less severe. | | | | | | | |

**Severity classification for antenatal corticosteroid studies**

| **Summary of animal model** | **Severity scoring (mild, moderate, severe)** | | | | | | |
| --- | --- | --- | --- | --- | --- | --- | --- |
|  | **Scorer 1** | **Scorer 2** | **Scorer 3** | **Scorer 4** | **Scorer 5** | **Scorer 6** | **Summary of scores** |
| In these studies antenatal corticosteroids are given to pregnant mothers prior to preterm delivery of their neonates, or to foetuses after delivery, or to foetuses in utero, or using a combination of methods. Mothers may have ultrasound scans and blood samples taken. They may be given antibiotics or progesterone. Some have abortions, some go into premature labour. The mothers are given caesarean sections to deliver their foetuses preterm. The foetuses are then removed from the mothers and either killed at delivery or observed for varying lengths of time (up to 6 days). Some foetuses are intubated, catheterised and mechanically ventilated after delivery. The animals used are rabbits, sheep, rats, monkeys, baboons, cows and their neonates.  Some foetuses were found dead in utero, or were stillborn. Many were found to be oedematous. Some died during the experimental period. After experiments foetuses were killed at different time points, including in utero / at delivery / after varying amounts of ventilation (from 15 mins to 24 hrs of ventilation) / left to die / at specified endpoints (from 5 minutes to 6 days).  Some mothers were killed either prior to delivery or around delivery, but the mothers’ fate was rarely reported. In one study baboon mothers were released back to gang cages after 4 weeks. | ***Mother:***  severe  ***Foetus:***  severe (but see later, if anaesthetised then Moderate or even Mild | ***Mother:*** Potentially severe  ***Foetus:*** potentially severe | ***Mother:*** Moderate  ***Foetus:*** Severe | ***Mother:***  Severe (for caesareans without adequate analgesic)  ***Foetus:***  Severe if survive beyond birth and have procedures without anaesthesia/ analgesia | ***Mother:***  Moderate if under anaesthesia during CS and drug administered with appropriate analgesia  ***Foetus:***  Severe (perhaps moderate if killed at birth). Could be moderate if no oedema and degree of prematurity is not too far from due date | ***Mother:***  Moderate to Severe  ***Foetus:***  Moderate to Severe | ***Mother***  Severe (1)  Potentially severe (1)  Severe if no analgesia (1)  Moderate (1)  Moderate if appropriate anaesthesia and analgesia used (1)  Moderate/ severe (1)  ***Foetus***  Severe (1)  Potentially severe (1)  Moderate/ severe (1)  Severe if: no anaesthesia 1; survives beyond birth and has no anaesthesia/ analgesia 1; survives beyond birth and very premature 1 (3) |
| **Method of drug administration:** Drugs may be delivered in the following ways: i. to pregnant mothers, ii. to the foetus after delivery, iii. to the foetus in utero, iv. to the foetus via their mother. A combination of these methods may be used. | | | | | | | |
| i. Mothers may have the drug either intramuscularly, sub-cutaneously, intravenously, or intraperitoneally (often over several weeks) prior to pre-term delivery. | ***Mother:***  Moderate | ***Mother:*** Moderate | ***Mother:*** Mild | ***Mother:***  Moderate | ***Mother:***  Moderate if drug administered with appropriate analgesia | ***Mother:***  Mild – moderate depending on outcome | ***Mother***  Moderate (3)  Moderate if analgesia used (1)  Mild (1)  Mild – moderate (1) |
| ii. For administration of drug to the foetus after delivery, the foetus has an endotracheal tube placed (not always with anaesthetic) through which to deliver the drugs. Upper airway leaks were reported during placement of endotracheal tubes in some studies. *Most foetuses have anaesthesia post-delivery, but some do not and* *in one study foetuses were paralysed and not anaesthetised*. Foetuses are given drugs either by injection or directly into trachea. Some may also be given fluids (IV, by clysis*, or by nasogastric tube). | ***Foetus:***  Severe – but if anaesthetised then Moderate or even Mild | ***Foetus:***  Severe | ***Foetus:***  Severe | ***Foetus:***  Severe (due to paralysis without anaesthesia) | ***Foetus:***  Severe when not anaesthetised | ***Foetus:***  Moderate but severe if no anaesthetic, if upper airway leaks, if paralysed and not anaesthetised | ***Foetus***  Severe (3)  Severe if not anaesthetised (3) (in which case potentially moderate) |
| iii. Drugs are administered to the foetus in utero either by injection, via laparotomy under anaesthesia, via intra-amniotic injection or via ultrasound-guided foetal injection. Some procedures involve additional ultrasounds and/ or amniocentesis. Sedation for the mother during these procedures was reported in only one study. | ***Foetus:***  Severe – but if anaesthetised then Moderate or even Mild  ***Mother:***  *Missing* | ***Foetus:*** *Missing*  ***Mother:***  Severe | ***Foetus:***  Mild  ***Mother:*** Moderate | ***Foetus:***  Mild if direct injection/ Nothing if intra-amniotic  ***Mother:***  Moderate / Severe (laparotomy) | ***Foetus:***  Severe when not anaesthetised  ***Mother:***  *Missing* | ***Foetus:***  Mild/ Moderate  ***Mother:***  Moderate / Severe | ***Foetus***  Missing (1)  Mild (1)  Mild if direct injection (1)  Mild/ moderate (1)  Severe if not anaesthetised (2)  ***Mother***  Missing (2)  Severe (1)  Moderate (1)  Moderate/ severe (2) |
| iv. For administration of the drug *via* mothers, pregnant mothers are given a hysterotomy under local anaesthesia. Catheters are placed in the foetuses (no anaesthesia reported), including tracheal catheters. The catheters pass from the uterus through an incision in the mother and are attached to her flanks. Drugs are delivered to the foetus via these catheters for up to 14 days. Some foetuses may have tracheal fluids withdrawn over days and weeks. | ***Mother:***  Moderate  ***Foetus:***  Severe – but if anaesthetised then Moderate or even Mild | ***Mother:*** Severe  ***Foetus:***  Severe | ***Mother:*** Moderate  ***Foetus:*** Moderate | ***Mother:***  Severe  ***Foetus:***  Moderate | ***Mother:***  Severe  ***Foetus:***  *Missing* | ***Mother:***  Moderate/ Severe  ***Foetus:***  Moderate/ Severe (Worst case - long) | ***Mother***  Moderate (2)  Severe (3)  Moderate/ severe (1)  ***Foetus***  Severe (1)  Moderate (2)  Moderate/ severe (1)  Missing (1)  Severe unless anaesthetised in which case moderate/ mild (1) |
| **Ventilation models** |  |  |  |  |  |  |  |
| For models using ventilation, foetuses have endotracheal tubes placed at pre-term delivery foetuses (anaesthesia and/or sedation for this rarely reported). Agents may be delivered via intra-tracheal instillations. Some have lung fluids aspirated.  Foetuses are placed on a mechanical ventilator for varying lengths of time, after which they are killed. Periods of mechanical ventilation ranged from 15 minutes to 24 hours. During ventilation many foetuses had pneumothoraces. Some suffered pulmonary interstitial emphysema.** Some foetuses were paralysed during ventilation and some were given pentobarbital to prevent spontaneous respiration. Some studies involved clamping the trachea during or after ventilation. Some foetuses had the endotracheal tube clamped.    Some foetuses also had catheters inserted and drugs and other fluids/ agents delivered through these (e.g. glucose, inulin, parenteral fluids, antibiotics) or blood taken. Some foetuses were given maternal blood. In one study radiolabelled microspheres were administered. | ***Foetus:***  if not anaesthetised then Severe. If anaesthetised the whole time then Moderate or Mild | ***Foetus:***  Severe | ***Foetus:*** Moderate | ***Foetus:***  Moderate (assuming under anaesthesia for ventilation etc.) | ***Foetus:***  Severe | ***Foetus:***  *Missing* | ***Foetus***  Severe (2)  Moderate (1)  Missing (1)  Moderate if anaesthetised (1)  Moderate/ mild if anaesthetised, otherwise severe (1) |
| ***Endpoints*** Please categorise the severity for each time period: | | | | | | | |
| In utero | Moderate | Moderate | Moderate | *Missing* | Moderate | Moderate | Moderate (5)  Missing (1) |
| At delivery | Moderate | Moderate | Moderate | Moderate | Moderate | Moderate | Moderate (6) |
| After 30 mins of age | Moderate | Severe | Moderate | *Missing* | Moderate | Moderate | Moderate (4)  Missing (1)  Severe (1) |
| After 2 hours of age | Severe | Severe | Moderate | Severe | Moderate | Severe | Severe (4)  Moderate (2) |
| After 6 hours of age | Severe | Severe | Moderate | Severe | Moderate | Severe | Severe (4)  Moderate (2) |
| At 1 -3 days of age | Severe | Severe | Moderate | Severe | Severe | Severe | Severe (5)  Moderate (1) |
| At 5 or 6 days of age | Severe | Severe | Moderate | Severe | *Missing* | Severe | Severe (4)  Missing (1)  Moderate (1) |
| Left to die with no endpoint | Severe | Severe | Moderate | Severe | Severe | Severe | Severe (5)  Moderate (1) |
| *After ventilation* |  |  |  |  |  |  |  |
| After 15 – 60 mins on ventilator | If not anaesthetised Severe, if anaesthetised Mild | Potentially severe | Moderate | Mild (if under anaesthesia) | Severe | Moderate | Moderate (2)  Severe (1)  Potentially severe (1)  Mild if anaesthetised , otherwise severe (2) |
| After 1-4 hours on ventilator | If not anaesthetised Severe, if anaesthetised Mild | Potentially severe | Moderate | Mild (if under anaesthesia) | Severe | Severe | Severe (2)  Potentially severe (1)  Moderate (1)  Mild if anaesthetised, otherwise severe (2) |
| After 24 hours on ventilator | If not anaesthetised Severe, if anaesthetised Mild | Potentially severe | Moderate | Mild (if under anaesthesia) | Severe | Severe | Severe (2)  Potentially severe (1)  Moderate (1)  Mild if anaesthetised, otherwise severe (2) |
| **Additional comments from scorers** |  |  |  |  |  | So much variation, hard to categorise. |  |
| **Overall summary** For the overall model scorers categorised harms to the mother as moderate to severe, depending on use of analgesia/ anaesthesia. For the overall model the scorers categorised harms to the foetus as generally severe, particularly if the foetus survived beyond birth and had no anaesthesia/ analgesia. For the administration of drugs to the mother scorers generally categorised this as moderate to mild. For the administration of drug to the foetus after delivery, scorers categorised this as severe, particularly if not anaesthetised. For administration of the drug to the foetus in utero scorers categorised the procedures to the foetus as mild, but severe if no anaesthesia was used and moderate to severe for the mother. For administration of the drug via the mother this was categorised as moderate to severe for the mother and moderate to severe for the foetus. For foetuses having mechanical ventilation after delivery scorers categorised this as severe, or moderate if anaesthesia was used. In terms of endpoints, scorers categorised harms for those killed in utero and at delivery as moderate; as moderate to severe for deaths post-delivery (30 mins to 6 days), with increasing severity scores as time post-delivery increased. Harms to those foetuses left to die with no endpoint were categorised as severe by 5/6 scorers (animals found dead should automatically score as ‘severe’). Foetuses having ventilation were scored as experiencing moderate to severe harms unless they were anaesthetised, with severity scores increasing with the amount of time spent on the ventilator. | | | | | | | |

*Clysis: introduction of large amounts of fluid into the body usually by parenteral injection. **Pulmonary interstitial emphysema (PIE) refers to the abnormal location of air within the pulmonary interstitium and lymphatics. Almost always associated with mechanical ventilation.

**Severity classification for thrombolysis studies**

| **Summary of animal model** | **Severity scoring (mild, moderate, severe)** | | | | | |  |
| --- | --- | --- | --- | --- | --- | --- | --- |
|  | **Scorer 1** | **Scorer 2** | **Scorer 3** | **Scorer 4** | **Scorer 5** | **Scorer 6** | **Summary of scores** |
| This model involves inducing a stroke in the animal and then testing the effect of a thrombolytic agent (clot buster) post stroke. Stroke may be surgically induced in a number of different ways, including flushing pre-fabricated blood clots into the carotid and cerebral arteries, blocking these arteries with filaments, tying the arteries or producing a thrombus with photo-illumination and photosensitive dye (Rose Bengal). The general approach is as follows:  On the day before surgery some animals may have undergone anaesthesia and preparatory surgery, e.g. to produce a clot, or place catheter, ligatures or probes. On day of experiment animals are anaesthetised and undergo surgery to induce a stroke using one of the above methods. During surgery they may also have their skulls opened for placement of probes and monitors. They will be given the thrombolytic agent (or saline and sometimes other agents) intravenously. They may have angiograms, CT scans or MRI scans whilst under anaesthesia. The use of paralytic agents was reported in 5 studies.  Upon recovery from anaesthesia animals may be observed for 2hrs to 24hrs, or observation can be extensive, over 1- 7 days, 2 weeks, or as much as 1 or 2 months, after which animals are killed. Large numbers of animals die before reaching the endpoints. Pre- or post-operative welfare mentioned in only 14% of studies (i.e. brief details about housing and access to food and water). The use of painkillers was reported in one study.  During the period of observation animals may be neurologically assessed, over 1-4hrs, 1-2 days, or for up to one week. Neurological assessments commonly involve being held upside down by the tail, resistance to lateral push etc. More detailed assessments may be conducted, e.g. seeing how long rats can remain on a horizontal suspended rotating rod, or how long it takes them to remove sticky tape from paws. Post stroke animals frequently had reduced levels of spontaneous activity, rapid involuntary movements of the eye, inability to stand, severely uncoordinated movements, hemiparesis.  Animals used: Rats (57 studies), rabbits (31 studies), mice (7 studies), guinea pigs, baboons, squirrel monkeys (1 study each) | Severe overall (profound effect on animal, low reporting of painkillers, high mortality) | Severe | Severe | Severe  (Studies that were brief, that didn’t open the skull, didn’t have severe effects (humane endpoints) and were given anaesthesia/ analgesia may score better | Severe  (due to the ‘severe impairment’ likely to be caused by the procedure plus the many tests of a ‘disabled’ animal. Induction of stroke per se may induce change in function, perhaps without conscious suffering in which case one might classify as Moderate if kept alive for a short time) but without knowing this, severe is the obvious / safest categorisation) | Short durations plus anaesthesia = Moderate  Longer durations Severe  Re some of the neuro assessments: Moderate to Severe | Severe (5)  Moderate to severe (1)  Some commented that if anaesthesia/ analgesia were used and duration of study brief, then studies might score less severely |
| **Variations of model** |  |  |  |  |  |  |  |
| Surgery under anaesthesia to place catheter, then recovery. Then stroke induced (via clot injected through catheter) whilst animals awake and restrained. Treatment with thrombolytic agent from 1hr to 24h post stroke, neurological assessment from 4 – 48hr post stroke. Killed between 1- 2 days, or at day 6 or 7 post stroke. | 2 procedures = Severe | Severe | Severe | Severe | Severe | Severe | Severe (6) |
| Surgery under anaesthesia to induce stroke, then recovery. Then treatment with thrombolytic agent up to 24h post stroke (occasionally re-anaesthetised for drug administration), neurological assessment within 24h of stroke, then killed up to 33h post stroke. | Might be Moderate if done ‘well’ | Severe | Severe | Severe | Severe | If anaesthesia throughout Moderate, otherwise Severe | Severe (4)  Moderate if anaesthesia used throughout, otherwise severe (1)  Moderate if ‘done well’ (1) |
| Surgery under anaesthesia to induce stroke, treatment with thrombolytic agent, then series of MRI scans whilst restrained in head holder and with ear bars. Anaesthesia usually maintained for scans. MRI scans for up to 8h post stroke, or at 1, 2 or 7 days. Killed after last MRI scan (up to 8h post stroke, or at 1, 2 or 7 days) | Multiple procedures = Severe | Severe | Severe | Severe | Severe (could be Moderate if under anaesthesia for nearly all the time and killed at 1 day) | Insufficient info on care and status of animal – Moderate to Severe | Severe (5)  Moderate to severe (1) |
| **Overall summary** In general scorers categorised the stroke model as severe. Some commented that if anaesthesia/ analgesia were used and duration of study brief, then studies might score less severely. Scorers categorised studies that induced stroke while animals were awake as severe. Most categorised relatively short term studies (up to 33h) that induced stroke under anaesthesia as severe. Most categorised studies that induced stroke under anaesthesia and then involved repeated MRI scans as severe. | | | | | | | |
